# Dilignans with a Chromanol Motif Discovered by Molecular Networking from the Stem Barks of *Magnolia obovata* and Their Proprotein Convertase Subtilisin/Kexin Type 9 Expression Inhibitory Activity

**DOI:** 10.3390/biom11030463

**Published:** 2021-03-19

**Authors:** Jongmin Ahn, Hee-Sung Chae, Pisey Pel, Young-Mi Kim, Young Hee Choi, Jinwoong Kim, Young-Won Chin

**Affiliations:** 1 College of Pharmacy and Research Institute of Pharmaceutical Sciences, Seoul National University, Seoul 08826, Korea; jmahn0205@gmail.com (J.A.); chaeheesung83@gmail.com (H.-S.C.); pisey.1603@gmail.com (P.P.); 0210121@hanmail.net (Y.-M.K.); jwkim@snu.ac.kr (J.K.); 2 College of Pharmacy and Integrated Research Institute for Drug Development, Dongguk University-Seoul, Goyang 10326, Korea; choiyh@dongguk.edu

**Keywords:** *Magnolia obovata*, Magnoliaceae, dilignans, molecular networking, PCSK9, lipid metabolism genes

## Abstract

Natural products have been fundamental materials in drug discovery. Traditional strategies for observing natural products with novel structure and/or biological activity are challenging due to large cost and time consumption. Implementation of the MS/MS-based molecular networking strategy with the in silico annotation tool is expected to expedite the dereplication of secondary metabolites. In this study, using this tool, two new dilignans with a 2-phenyl-3-chromanol motif, obovatolins A (**1**) and B (**2**), were discovered from the stem barks of *Magnolia obovata* Thunb. along with six known compounds (**3**–**8**), expanding chemical diversity of lignan skeletons in this natural source. Their structures and configurations were elucidated using spectroscopic data. All isolates were evaluated for their PCSK9 mRNA expression inhibitory activity. Obovatolins A (**1**) and B (**2**), and magnolol (**3**) showed potent lipid controlling activities. To identify transcriptionally controlled genes by **1** along with downregulation of PCSK9, using small set of genes (42 genes) related to lipid metabolism selected from the database, focused bioinformatic analysis was carried out. As a result, it showed the correlations between gene expression under presence of **1**, which led to detailed insight of the lipid metabolism caused by **1**.

## 1. Introduction

Natural products have been considered important sources for drug discovery and development [1]. Conventional strategies (i.e., bioassay-guided isolation) to discover bioactive substances from natural products demand large cost and time consumption (i.e., laborious isolation and identification process of active substances, re-isolation of the same active substances). To overcome one of these drawbacks, various dereplication and structure identification methods have been developed [2]. Among these methods, liquid chromatography tandem mass spectrometry (LC-MS/MS) is the common technology for comprehensive profiling of natural products and helps facilitate structural characterization of constituents in complex mixtures [3,4,5]. Molecular networking (MN) is one of the recent bioinformatics approaches that estimate structural similarity by comparing MS/MS data [6,7]. Recently, this tool has been increasingly used in natural product discovery, which enables to find candidate active molecules directly from fractionated bioactive extracts [8], portray the taxonomic relationship between different plant species [9], or depict an overview on chemical diversity between a plant’s different parts [3]. Furthermore, computational MS/MS data analysis through in silico annotation tools can improve capturing and inferring structural information from MS/MS data [9,10,11]. Using these powerful tools superior to traditional dereplication strategies, we could identify analogues with chemical novelty and prioritize secondary metabolites for isolation. The aim of this study was to describe an MS/MS-based molecular networking strategy with the in silico annotation tools that are expected to facilitate the dereplication of secondary metabolites, based on the natural products as fundamental materials in drug discovery.

Excessive levels of low-density lipoprotein cholesterol (LDL-C) can lead to atherosclerotic cardiovascular diseases. Lowering of LDL-C in the plasma has been one strategy for its prevention and treatment [12]. Proprotein convertase subtilisin/kexin type 9 (PCSK9) binds to low-density lipoprotein receptor (LDLR) and forms PCSK9-LDLR complex, which leads to the degradation of LDLR in lysosomes and resultantly increases circulating LDL-C [13]. Thus, inhibition of PCSK9 has been suggested as a new therapeutic target to downregulate LDL-C in the plasma [14]. Recently, various small molecules from natural sources have been reported to modulate PCSK9 expression [15,16,17,18,19]. By PCSK9 mRNA expression monitoring assay in HepG2 cell lines for botanical extracts, the *n*-hexane extracts of the dried stem bark of *Magnolia obovata* was found to inhibit PCSK9 mRNA expression.

*M. obovata* Thunb. (Magnoliaceae), a deciduous tree distributed in East Asia, has been traditionally used for the treatment of various disorders (e.g., gastrointestinal disorders, fever, anxiety and allergic diseases) in Northeast Asia [20,21,22]. Neolignans, particularly honokiol, magnolol, and obovatol, have been reported as the main substances responsible for various biological activities [23,24]. Additionally, diverse lignan derivatives (e.g., sesquiterpene-neolignans and dilignans) derived from major neolignans (magnolol and obovatol) have been discovered in *M. obovata* [22,24,25,26]. Herein, to expand the chemical diversity of lignan derivatives in this natural source, an MS/MS-based MN strategy using the Global Natural Products Social Molecular Networking (GNPS) web platform (https://gnps.ucsd.edu/ProteoSAFe/status.jsp?task=3a3d98903cbc4cc5bbb214fa0c7d1ce4, accessed on 19 March 2021) [27] with in silico annotation via Network Annotation Propagation (NAP) was applied (https://proteomics2.ucsd.edu/ProteoSAFe/status.jsp?task=680053703c2b49f7ab438882c6badab1, accessed on 19 March 2021)) [28]. In addition, all compounds isolated from the present study were assessed for their PCSK9 expression inhibitory activities. To further understand active compound’s effects on lipid metabolism, correlations between lipid metabolism-related gene expression caused by the active compound were assessed and their protein-protein interactions were depicted.

## 2. Materials and Methods

### 2.1. General Experimental Procedures

Optical rotations were obtained with a JASCO P-2000 digital polarimeter (JASCO, Tokyo, Japan). UV and ECD spectra were measured with a Chirascan plus circular dichroism spectrometer (APL, Leatherhead, Surrey, UK). IR spectra were recorded on a JASCO FT/IR-4200 spectrophotometer (JASCO, Tokyo, Japan). 1D (^1^H and ^13^C) and 2D (^1^H-^1^H COSY, HSQC, and HMBC) NMR spectra were obtained with a Bruker AVANCE-600. The ^1^H-^1^H ROESY NMR spectra were obtained using Bruker AVANCE III HD 850 MHz spectrometer equipped with 5 mm TCI cryoprobe (Bruker, Billerica, MA, USA). Methanol-*d_4_* (Cambridge Isotope Laboratories, Inc., Cambridge, MA, USA) were used as NMR solvents and reference peaks (δ_H_ 3.31 and δ_C_ 49.0). High resolution-electrospray ionization-mass spectroscopy (HRESIMS) and MS/MS data were obtained using a Waters Xevo G2 Q-TOF mass spectrometer (Waters, Milford, MA, USA). Purifications were performed using MPLC with a Combiflash Companion (Isco, Lincoln, NE, USA) and HPLC with a Gilson 321 pump and Gilson 172 diode array detector (Gilson, Middleton, WI, USA). HPLC columns used for purification were 250 × 10 mm Luna 5 μm C18 100 Å (Phenomenex, Seoul, Korea), 250 × 10 mm YMC-Pack Ph 5 μm 120 Å (YMC Co., Ltd., Kyoto, Japan), and 250 × 21 mm Synergi™ 4 µm Hydro-RP 80 Å (Phenomenex, Seoul, Korea). HPLC grade solvents were purchased from Fisher Scientific Korea Ltd. (Seoul, Korea). Water for HPLC was purified using a Milli-Q system (Millipore, Milford, MA, USA). Solvents for isolation were purchased from Daejung Chemical & Metal Co., Ltd. (Siheung, Korea).

### 2.2. Plant Material

The dried bark of *Magnolia obovata* Thunberg (Magnoliaceae) was collected from Mokpo, Jeollanam-do in June 2017, and identified by Dr. H-S. Chae. A voucher specimen (CYWSNU-KP0018) has been deposited at the medicinal plant garden in Seoul National University.

### 2.3. MS/MS Molecular Networking

The sub-fractions of *n*-hexane extract were analyzed with a Waters Acquity UPLC BEH C18 column (150 mm × 2.1 mm, 1.7 µm, 40 °C) using the Waters Acquity UHPLC-QTOF-MS/MS system (Waters Co., Milford, MA, USA). The mobile phase was water (A) and acetonitrile (B), with the following gradient: 30–95% B (0–20 min, *v*/*v*), 95% B (20–23 min, *v*/*v*), and 100% (23–27 min, *v*/*v*). The flow rate was 300 µL/min, and the injection volume was 2.0 µL. The MS experiments were performed in data-dependent acquisition (DDA) mode through an electrospray ionization (ESI) condition: negative ion mode, capillary voltage 2.9 kV, cone voltage 40 V, source temperature 120 °C, desolvation gas temperature 350 °C, cone gas flow 50 L/h, and desolvation gas flow 800 L/h. The full MS survey scan was performed for 150 ms in the range of 100–1500 Da. The MS/MS data were converted to the .mzXML format using MSConvert and then uploaded on the GNPS web platform (https://gnps.ucsd.edu, accessed on 19 March 2021) for molecular networking. NAP workflow (https://proteomics2.ucsd.edu/ProteoSAFe/?params={%22workflow%22:%22NAP_CCMS2%22}, accessed on 19 March 2021) was performed for in silico analysis. All the results and parameters can be accessed with the GNPS job identification for molecular network analysis (https://gnps.ucsd.edu/ProteoSAFe/status.jsp?task=3a3d98903cbc4cc5bbb214fa0c7d1ce4, accessed on 19 March 2021) and NAP in silico analysis (https://proteomics2.ucsd.edu/ProteoSAFe/status.jsp?task=680053703c2b49f7ab438882c6badab1, accessed on 19 March 2021).

### 2.4. Extraction and Isolation

Dried barks of *M. obovata* (3.0 kg) were extracted with 94% ethanol (10 L) under sonication for 90 min (three times) at room temperature. After removal of the solvent *in vacuo*, crude extract (464.5 g) was partitioned with *n*-hexane and water. The *n*-hexane fraction (158.6 g) was subjected to silica gel MPLC using gradient mixtures of *n*-hexane and EtOAc (100:0 → 0:100) to yield 11 fractions (H01–H11). H06 (648.3 mg) was separated using RP silica gel MPLC eluted with aq. CH_3_CN (30% → 100%) to 13 subfractions (H06a–H06m). H06i (40.1 mg) was further purified using semipreparative HPLC Luna column (CH_3_CN–H_2_O, 35:65, 2.5 mL/min, 210 nm) to give **1** (6.2 mg, t_R_ 43.1 min) and **2** (4.1 mg, t_R_ 36.2 min). H03 (72.6 g) was fractionated using silica gel MPLC eluted with *n*-hexane−ethylacetate (20:1 → 0:100) to obtain 4 subfractions (H03a–H03d). Compounds **3** (91.0 mg, t_R_ 23.9 min), **4** (18.2 mg, t_R_ 22.7 min), and **5** (15.1 mg, t_R_ 19.0 min) were purified from a portion of H03b by preparative HPLC (Synergi column, CH_3_CN–H_2_O, 75:25, 5 mL/min, 240 nm). H09 (855.9 mg) was fractionated by RP silica MPLC with aq. methanol (10% → 100%) to afford **7** (2.1 mg, H09i), **8** (3.6 mg, H09g), and 13 subfractions (H09a–H09p). H09h (25.6 mg) was further purified by semipreparative HPLC (YMC column, CH_3_CN–H_2_O, 40:60, 3 mL/min, 254 nm) to give **6** (6.3 mg, t_R_ 22.4 min).

Obovatolin A (**1**): pale yellow, amorphous solid; ${\left[\alpha \right]}_{D}^{20}$ −18.9 (*c* 0.1, CH_3_OH); UV (CH_3_OH) λ_max_ (log ε) 276 (1.67), 239 (2.37) nm; ECD (CH_3_OH) λ_max_ (Δ ε) 282 (−0.22), 259 (−0.24), 212 (+0.83); IR (neat) ν_max_ 3327, 2979, 2922, 1603, 1504, 1449, 1318, 1218, 1054, 1033, 1015 cm^−1^; ^1^H and ^13^C NMR data, see Table 1; HRESIMS *m*/*z* 577.2233 [M − H]^−^ (calcd. for C_36_H_33_O_7_, 577.2226).

Obovatolin B (**2**): pale yellow, amorphous solid; ${\left[\alpha \right]}_{D}^{20}$ 169.4 (*c* 0.1, MeOH); UV (CH_3_OH) λ_max_ (log ε) 284 (1.56), 233 (2.34) nm; ECD (CH_3_OH) λ_max_ (Δ ε) 293 (−0.19), 273 (−0.20), 250 (−0.22), 221 (+0.77), 210 (+0.85); IR (neat) ν_max_ 3363, 2930, 1639, 1603, 1504, 1434, 1218, 1033, 1017, 917 cm^−1^; ^1^H and ^13^C NMR data, see Table 1; HRESIMS *m*/*z* 561.2257 [M − H]^−^ (calcd. for C_36_H_33_O_6_, 561.2277).

### 2.5. Cell Culture, Drugs and Chemicals

The HepG2 human hepatocellular liver cell line was purchased from the Korea Research Institute of Bioscience and Biotechnology (Daejeon, Korea). These cells were grown in Eagle’s Minimum Essential Medium (EMEM) containing 10% fetal bovine serum with 100 U/mL penicillin/streptomycin sulfate. Cells were incubated in a humidified 5% CO_2_ atmosphere at 37 °C. EMEM, penicillin, and streptomycin were purchased from Hyclone (Logan, UT, USA). Bovine serum albumin was purchased from Sigma-Aldrich (St. Louis, MO, USA).

### 2.6. Quantitative Real-Time RT-PCR

A Trizol RNA extraction kit was used for isolation of total cellular RNA according to the manufacturer’s instructions. Briefly, for cDNA synthesis from RNA, total RNA (1 μg) was treated with 200 units reverse transcriptase and 500 ng oligo-dT primers in 50 mM Tris-HCl (pH 8.3), 75 mM KCl, 3 mM MgCl_2_, 10 mM dithiothreitol, and 1 mM dNTPs at 42 °C for 1 h. The reaction was quenched with incubating the solution at 70 °C for 15 min. PCR reactions were conducted using 1 μL cDNA and 9 μL master mix containing TB Green™ Premix Ex Taq™ II (Takara, Kyoto, Japan), 5 pmol of forward primer, and 5 pmol reverse primer, in a CFX384 Real-Time PCR Detection System (Bio-Rad, Hercules, CA, USA). The specificity of the amplification was confirmed by a melting curve analysis. CFX Manager Software (Bio-Rad) was used for data collecting and recording as a function of the threshold cycle (C_T_). The relative quantity of the controlled gene was normalized compared to the relative quantity of GAPDH (ΔΔCT). The equation 2^−(ΔΔCT)^ was used for calculating the mRNA abundance. Gene-specific primers were custom-synthesized by Bioneer (Daejeon, Korea). The specific primer sets are shown in Table S1.

### 2.7. Immunoblot Analysis

Western blot was carried out according to standard procedures [29]. Images were acquired using a ChemiDoc Imaging system (ChemiDoc™ XRS system with Image Lab™ software 3.0; Bio-Rad, Hercules, CA, USA).

### 2.8. Statistical Analysis

For multiple comparisons, one-way analysis of variance (ANOVA) was performed followed by Dunnett’s *t*-test. Fold changes and statistic calculations were performed using BIO-RAD CFX Maestro 1.1 software. Data from experiments are presented as means ± standard error of the mean (SEM).

### 2.9. Selection of Candidate Genes and Construction of Protein-Protein Interaction Network

The protein-protein interaction networks of PCSK9 were constructed using online database resource Search Tool for the Retrieval of Interacting Genes (STRING) (http://string-db.org/, accessed on 19 March 2021) [30] and visualized using Cytoscape software. In this study, STRING was used for interactions among proteins encoded by candidate genes, which was significantly changed by 1. A *p*-value < 0.05 was set as the cut-off criterion.

### 2.10. ADMET (Absorption, Distribution, Metabolism, Excretion and Toxicity) Analysis

The active compound **1** was subjected to ADMET property calculation using the chemoinformatic tool, pkCSM [31]. The SMILES of the compound was submitted to pkCSM web server (http://biosig.unimelb.edu.au/pkcsm/prediction_single/adme_1615762956.82, accessed on 19 March 2021) and its pharmacokinetic properties were predicted [32].

## 3. Results and Discussion

The methanol extract of *M. obovata* barks was partitioned using *n*-hexane, chloroform, ethyl acetate, *n*-butanol, and water. Through preliminary bioassay, the *n*-hexane fraction was found to inhibit PCSK9 mRNA expression (Figure S1). The *n*-hexane fraction was further fractionated by a silica gel column chromatography to yield 11 subfractions, and then these subfractions were analyzed by UHPLC-Q-ToF-MS/MS (negative-ion mode).

### 3.1. Dereplication Using GNPS Molecular Networking and Network Annotation Propagation (NAP)

**Figure 1:**
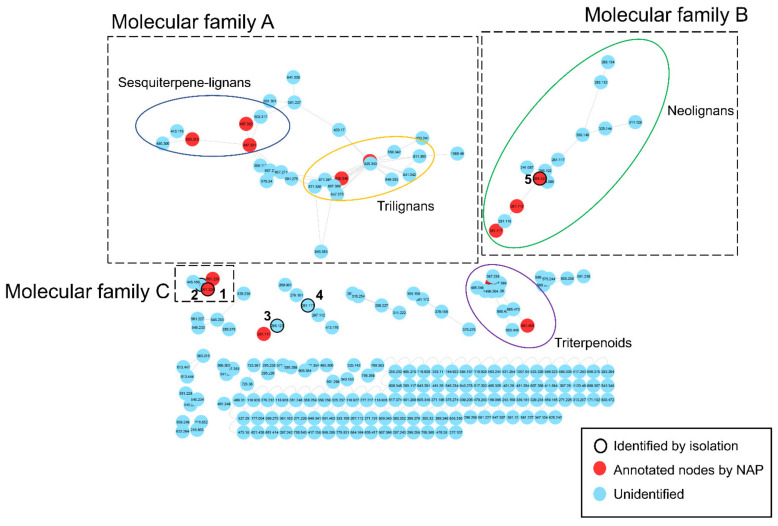
The MS/MS molecular network from the LC-MS/MS data of the *n*-hexane fraction of *M. obovata* barks.

To reveal chemical diversity of *n*-hexane fraction, the MS/MS data of subfractions were analyzed by the GNPS MS/MS molecular networking. In the network, nodes are indicative of a consensus MS spectra and edges represent spectral similarity between any two given nodes [10]. The resulting MS/MS molecular networking depicted constituents of the *n*-hexane fraction as 208 nodes, consisting of 23 molecular families (MFs) and 97 singletons (single nodes without any correlation) [3] (Figure 1). Most of molecular families were estimated to have lignan moieties based on in silico annotation *via* Network Annotation Propagation (NAP) module in GNPS (Figure S2). The molecular families A–C represented lignans made from allylphenyl groups. MF A was a big cluster composed of nodes with *m/z* 300–1100, which was further subdivided into sesquiterpene-lignans or tri-lignans classes. MF B represented neolignans consisting of two allylphenyl monomers with *m/z* 240–330 molecular weight ranges. A small cluster MF C was annotated to be dilignans made from allylphenyl moieties by analysis of MS/MS data and NAP module. Compounds in MF C were subjected to be isolated for discovering new dilignans and affirming robustness of the dereplication tool used.

### 3.2. Isolation and Structural Characterization

**Figure 2:**
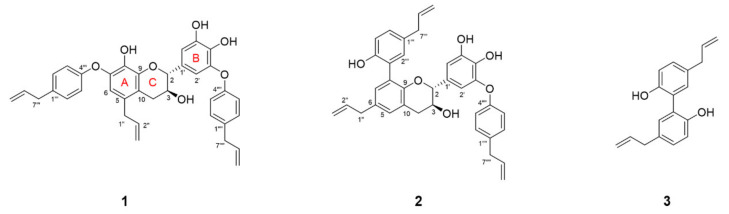
New compounds (**1**, **2**) and active magnolol (**3**).

Two new dilignans with a 2-phenyl-3-chromanol motif (**1** and **2**, Figure 2) made from allylphenyl groups were isolated from stem barks of *M. obovata* by targeted isolation using the LC-MS/MS-based molecular networking. In addition, the known neolignans composed of two allylphenol, magnolol (**3**) [33], obovatol (**4**) [33], and honokiol (**5**) [33], along with known (−)-syringaresinol (**6**) [33], (+)-pinoresinol (**7**) [34], and (−)-demethoxypinoresinol (**8**) [35] were purified and identified by comparison of their spectroscopic data and literatures.

**Figure 3:**
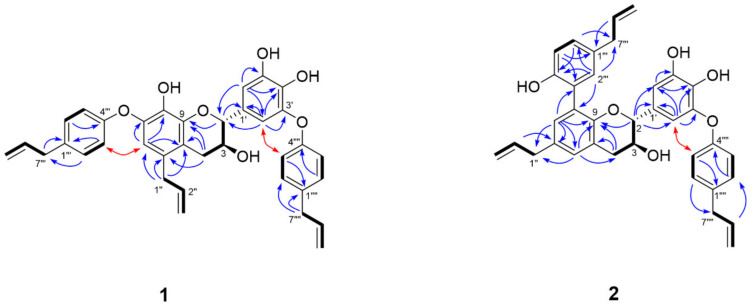
^1^H-^1^H COSY (thick black line), key HMBC (blue arrow), and ROESY (red arrow) correlations of compounds **1** and **2**.

Obovatolin A (**1**) was obtained as a pale yellow, amorphous solid. Its molecular formula was deduced to be C_36_H_34_O_7_ by the observed deprotonated molecule at *m/z* 577.2233 [M − H]^−^ (calcd. for C_36_H_33_O_7_, 577.2226). Two 1,4-disubstituted aromatic rings at δ_H_ 7.08 (2H, d, *J* = 8.6 Hz, H-2″″, 6″″), 7.06 (2H, d, *J* = 8.6 Hz, H-2‴, 6‴), 6.86 (2H, d, *J* = 8.6 Hz, H-3″″, 5″″), and 6.78 (2H, d, *J* = 8.6 Hz, H-3‴, 5‴), one pair of *meta*-coupled aromatic protons due to a 1,3,4,5-tetrasubstituted benzene ring (B) at δ_H_ 6.71 (1H, d, *J* = 1.9 Hz, H-6′), and 6.46 (1H, d, *J* = 1.9 Hz, H-2′), and one singlet aromatic proton at δ_H_ 6.34 (1H, s, H-6) assignable to a 1,2,3,4,5-pentasubstituted benzene ring (A), were observed in the ^1^H NMR spectrum (Table 1), along with the COSY cross peaks between H-2′ and H-6′, between H-2‴ and H-3‴, and between H-2″″ and H-3″″ (Figure 3). Additionally, the presence of three allyl groups was confirmed by ^1^H-^1^H COSY correlations of δ_H_ 5.84 (1H, m, H-2″) with δ_H_ 3.19 (2H, d, *J* = 6.2 Hz, H-1″) and 4.92 (2H, m, H-3″), and of δ_H_ 6.00–5.88 (2H, overlap, H-8‴ and 8″″) with δ_H_ 3.32 (4H, overlap, H-7‴ and 7″″) and 5.07–4.95 (4H, overlap, H-9‴ and 9″″). The remaining ^1^H NMR signals were attributed to the spin system of -CH(O)-CH(O)-CH_2_- in **1**, which was confirmed by the COSY correlations of δ_H_ 4.10 (1H, m, H-3) with δ_H_ 4.80 (1H, d, *J* = 6.3 Hz, H-2), 2.83 (1H, dd, *J* = 16.4, 5.0 Hz, H-4a), and 2.66 (1H, dd, *J* = 16.4, 6.8 Hz, H-4b). Based on these data, it was suggested that the structure **1** is a dilignan composed of four phenylpropanoids. The ^13^C NMR and HSQC spectra showed quaternary aromatic carbon signals that include eight oxygenated carbons at δ_C_ 158.1 (C-4‴), 157.5 (C-4″″), 148.1 (C-5′), 145.6 (C-3′), 144.6 (C-9), 143.0 (C-7), 138.0 (C-4′), 136.9 (C-8), and five non-protonated carbons at δ_C_ 135.5 (C-1″″), 134.9 (C-1‴), 131.3 (C-1′), 130.2 (C-5) and 116.8 (C-10).

The HMBC correlations from H-2″″ to C-7″″, from H-6 to C-1″, and from H-2‴ to C-7‴ enabled to locate three allyl groups to each benzene ring. Further HMBC correlations from H-3‴ and H-7‴ to C-1‴, from H-2‴ to C-4‴, from H-3″″ and H-7″″ to C-1″″, and from H-2″″ to C-4″″ were indicative of the presence of two 4-allylphenol groups. Additionally, the HMBC spectrum showed the correlations of H-1″ with C-5, C-6 and C-10, and of H-6 with C-7, C-8 and C-10, suggesting the remaining allyl group was attached to the A ring. The assignment of the 1,3,4,5-tetrasubstituted benzene ring (B) was deduced by the HMBC cross peaks of H-2′ with C-1′, C-3′ and C-4′, and of H-6′ with C-4′ and C-5′. The chromanol moiety C-ring [-CH(O)-CH(O)-CH_2_-] in **1** was supported by the HMBC correlations from H-2 to C-9 and C-1′, from H-2′ and H-6′ to C-2, and from H-4 to C-5, C-9 and C-10. Two 4-allylphenol groups were connected *via* ether linkages at C-7 and C-3′, respectively, confirmed by the ROESY correlations of H-6 with H-3‴, and of H-2′ with H-3″″. The *trans* configuration between H-2 and H-3 was verified by the coupling constant (*J_2,3_* = 6.3 Hz) [36]. The absolute configuration was determined to be 2*R* and 3*S* on the basis of the ECD spectrum, which showed negative Cotton effect at 275 nm [37]. Therefore, the structure of **1** was deduced as (2*R*,3*S*)-5-allyl-7-(4-allylphenoxy)-2-(3-(4-allylphenoxy)-4,5-dihydroxyphenyl)chromane-3,8-diol, a dilignan derived from two units of obovatol.

Obovatolin B (**2**) was isolated as pale yellow, amorphous solid. The molecular formula, C_36_H_34_O_6_, was determined based on HRESIMS (*m/z* 561.2257 [M − H]^−^; calcd. for C_36_H_33_O_6_, 561.2277) and NMR data analysis. Two 1,3,4,5-tetrasubstituted benzene rings at δ_H_ 6.36 (1H, d, *J* = 2.2 Hz, H-2′), 6.61 (1H, d, *J* = 2.2 Hz, H-6′), 6.84 (1H, d, *J* = 2.2 Hz, H-7), and 6.88 (1H, d, *J* = 2.2 Hz, H-5), one 1,4-disubstituted aromatic ring at δ_H_ 6.76 (2H, d, *J* = 8.5 Hz, H-3″″, 5″″), and 7.04 (2H, d, *J* = 8.5 Hz, H-2″″, 6″″), one 1,3,4-trisubstituted benzene ring at δ_H_ 6.70 (1H, d, *J* = 2.0 Hz, H-2‴), 6.72 (1H, d, *J* = 8.2 Hz, H-5‴), and 6.91 (1H, dd, *J* = 8.2, 2.0 Hz, H-6‴) were observed in the ^1^H NMR spectroscopic data along with the COSY correlations of H-5 with H-7, H-2′ with H-6′, H-5‴ with H-6‴, and H-2″″ with H-3″″ (Figure 3). Additionally, three allyl groups were deduced by ^1^H-^1^H COSY correlations of δ_H_ 5.89 (1H, m, H-8‴) with δ_H_ 3.20 (2H, d, *J* = 6.8 Hz, H-7‴) and 4.98 (2H, m, H-9‴), and of δ_H_ 5.96-5.95 (2H, overlap, H-2″ and 8″″) with δ_H_ 3.33 (4H, overlap, H-1″ and 7″″) and 5.05-5.04 (4H, overlap, H-3″ and 9″″). Sequential COSY correlations of H-2 [δ_H_ 4.79 (1H, d, *J* = 5.6 Hz, H-2)]/H-3 [δ_H_ 4.07 (1H, m, H-3)]/H-4 [(2.91 (1H, dd, *J* = 16.4, 4.7 Hz, H-4a) and 2.77 (1H, dd, *J* = 16.4, 6.3 Hz, H-4b)] indicated the presence of the spin system of -CH(O)-CH(O)-CH_2_- in **2**.

The ^13^C NMR and HSQC spectra displayed quaternary aromatic carbon signals that include six oxygenated carbons at δ_C_ 157.4 (C-4″″), 153.7 (C-4‴), 150.6 (C-9), 147.9 (C-5′), 145.3 (C-3′) and 137.6 (C-4′), and seven non-oxygenated carbons at δ_C_ 135.3 (C-1″″), 133.1 (C-6), 132.1 (C-1‴), 131.6 (C-1′), 127.7 (C-8), 127.0 (C-3‴) and 121.5 (C-10). A 5-substituted magnolol moiety was confirmed by the HMBC spectrum which showed the cross spots of H-5 with C-9 and C-1″, of H-7 with C-6, C-9, C-1″ and C-3‴, of H-2‴ with C-8, C-4‴ and C-7‴, of H-5‴ with C-1‴ and C-3‴, and of H-6‴ with C-4‴ and C-7‴. The HMBC correlations of H-2″″ with C-4″″ and C-7″″, and of H-3″″ with C-1″″ confirmed presence of a remaining 4-allylphenol group, which was attached to B ring via ether linkage at C-3′, supported by the ROESY correlations between H-3″″ and H-2′.

The chromanol moiety C-ring [-CH(O)-CH(O)-CH_2_-] in **2** was supported by the HMBC correlations from H-2 to C-9, C-1′, C-2′ and C-6′, from H-4 to C-9 and C-10, and from H-5 to C-4. The relative and absolute configurations of **2** were determined to be identical to those of **1** from its coupling constant between H-2 and H-3 (*J_2,3_* = 5.6 Hz) and its ECD spectrum. These spectroscopic data supported the structure of **2** as (2*R*,3*S*)-6-allyl-8-(5-allyl-2-hydroxyphenyl)-2-(3-(4-allylphenoxy)-4,5-dihydroxyphenyl)chromane-3-ol, a dilignan composed of one obovatol and one magnolol.

### 3.3. Biological Assay for PCSK9 mRNA Inhibitory Effect

**Figure 4:**
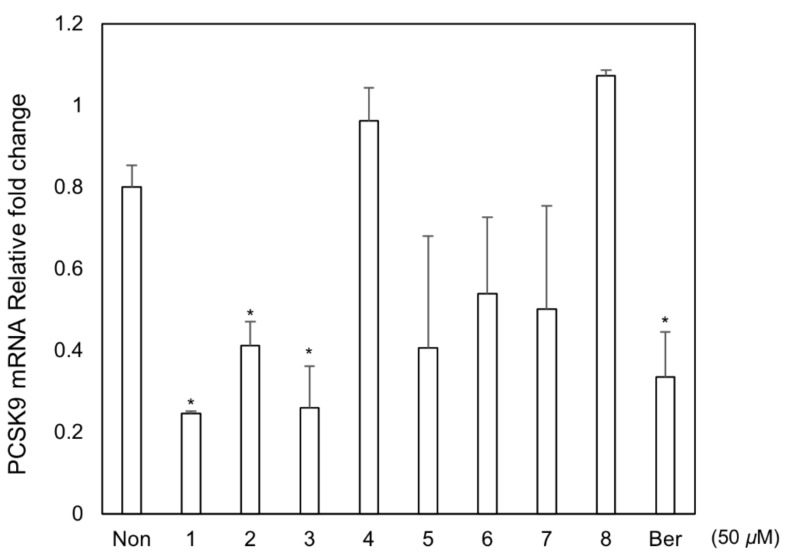
Effect of isolates from *M. obovata* on PCSK9 in the HepG2 human hepatocellular liver carcinoma cell line. Expression of PCSK9 mRNA was assayed by qRT-PCR in cells treated with each compound (50 μM), or positive control, berberine (Ber, 50 μM) for 24 h. * *p* < 0.05.

**Figure 5:**
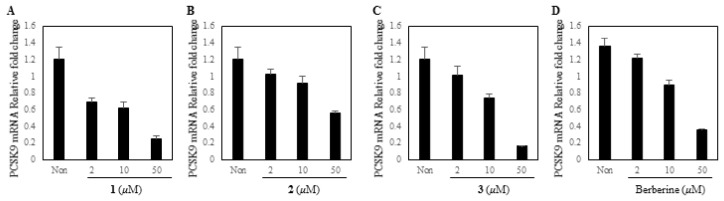
Effect of active compounds on PCSK9 in the HepG2 human hepatocellular liver carcinoma cell line. PCSK9 mRNA inhibitory effects of **1** (**A**), **2** (**B**), and magnolol (**3**) (**C**) were comparable with positive control, berberine (**D**).

All the isolates **1**–**8** were tested in PCSK9 mRNA expression using HepG2 cell (Figure 4). Out of them, **1**, **2**, and magnolol (**3**) were found to inhibit PCSK9 mRNA expression with IC_50_ values of 12.0, 45.4 and 22.9 µM (berberine IC_50_ 13.3 µM), respectively (Figure 5), while other compounds deemed inactive. These data suggested that new compounds **1**, **2**, and a major constituent, magnolol (**3**) from the stem barks of *M. obovata* could potentially increase cellular LDL-cholesterol uptakes in the plasma through inhibition of PCSK9 expression. Of active compounds, compound **1**, the most potent inhibitor of PCSK9 mRNA expression, was further tested for additional lipid metabolism-related gene expressions analysis.

### 3.4. Construction of Protein-Protein Interaction Network of Candidate Genes

**Figure 6:**
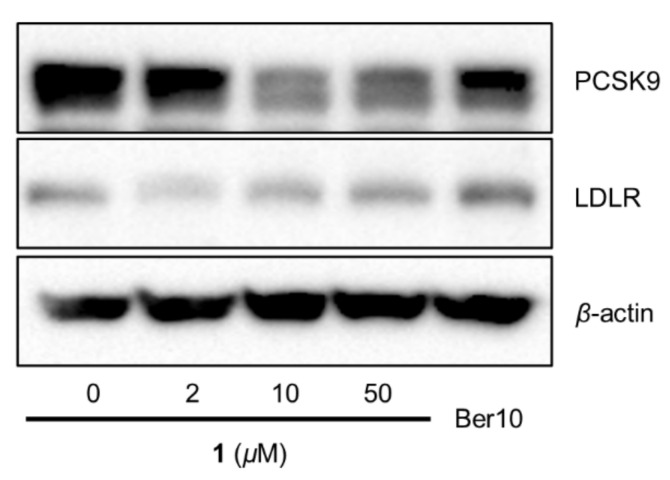
Effects of **1** on PCSK9 and LDLR expressions in the HepG2 human hepatocellular liver carcinoma cell line. Expressions of PCSK9 and LDLR were assayed by Western blot in cells treated with **1** and berberine (Ber, 10 μM) for 24 h.

Further Western blotting analysis showed that **1** decreased PCSK9 protein levels in HepG2 cells as well as increased LDLR expression as comparable to berberine (positive control) (Figure 6).

**Figure 7:**
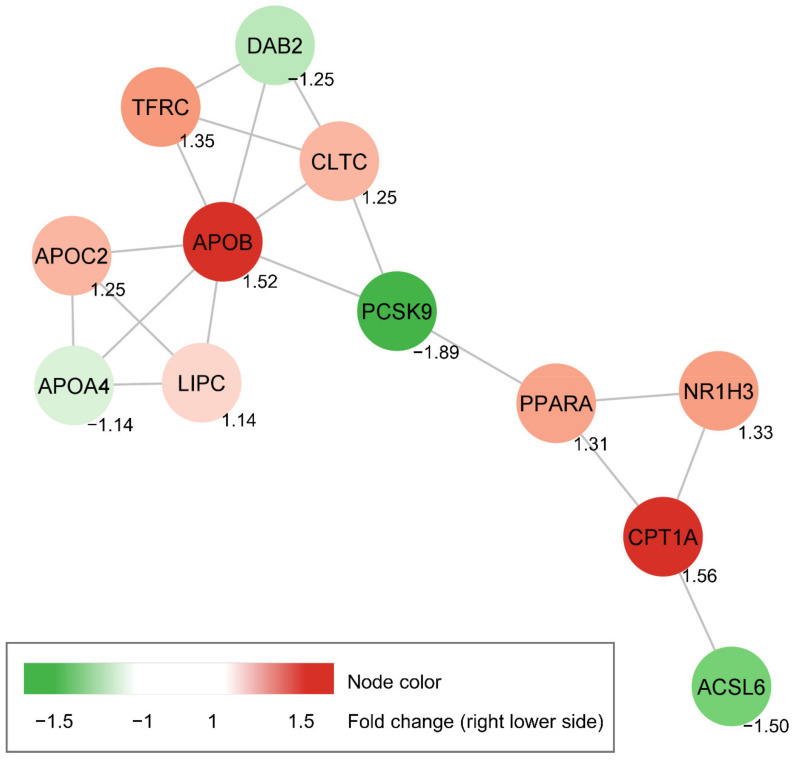
The protein-protein interaction network of the PCSK9-related genes significantly changed by **1**. PCSK9-related gene profiling of HepG2 cells treated with **1** for 24 h.

Small set of lipid metabolism-related genes (42 genes) were selected from the STRING database, and their expressions caused by **1** were tested (Table S1). As a result, the genes significantly sensitive to the treatment of **1** were observed and the regulated genes were chosen based on *p*-value to generate the interaction network. Further, the STRING database was used to analyze the interaction of those genes. The protein-protein interaction network (Figure 7) indicated that PCSK9 downregulation by **1** might be associated with upregulation of nuclear receptor subfamily 1 group H member 3 (NR1H3), also known as LXRα (Liver X receptor α), and peroxisome proliferator activated receptor α (PPARA) genes expression. Additionally, downstream target genes of transcriptional factors such as Acyl-CoA synthetase long-chain family 6 (ACSL6), carnitine palmitoyl transferase 1A (CPT1A), and apolipoprotein B (APOB) were upregulated or downregulated. The ACSL6, CPT1A, and APOB genes control fatty acid metabolism, triglyceride metabolism, and catabolism of lipoprotein particles, respectively [38,39,40].

### 3.5. ADMET Studies

The pharmacokinetic properties of **1** were calculated by the pkCSM and the data are shown in Figure S19. According to the predicted pharmacokinetic properties, **1** is poorly soluble in water but has great human intestinal absorption (87% ability). It has the steady state volume of distribution (VDss) value of −1.251, which indicates this compound may be distributed more in blood plasma than in tissues [41]. This compound **1** is also considered to be cytochrome P 450 enzymes (CYP) inhibitors such as CYP2C9 inhibitor, and CYP3A4 inhibitor. Cytochrome P 450 enzymes (CYP) metabolize many statins used for cholesterol-lowering drugs. CYP3A4 metabolizes atorvastatin and lovastatin and CYP2C9 metabolizes rosuvastatin and fluvastatin [42,43]. Thus, compound **1** has potential drug interaction with specific statins.

## 4. Conclusions

Implementation of MN strategy with the in silico annotation tools in natural product discovery is expected to expedite the dereplication of several secondary metabolites. Two new dilignans with a 2-phenyl-3-chromanol motif, obovatolins A (**1**) and B (**2**), were identified using these tools, resulting in an expansion of the chemical diversity with lignan skeleton in *M. obovata*. This study also suggested that new compounds (**1**, **2**), and a major magnolol (**3**) have potential of decreasing LDL-C in the plasma through inhibition of PCSK9 expression. In addition, the results herein showed correlations between lipid metabolism-related gene expressions under presence of **1**, which may help understand the lipid metabolism caused by **1**.

## Figures and Tables

**Table 1:** NMR spectroscopic data (800 MHz, methanol-d_4_) for obovatolins A (**1**) and B (**2**).

	Obovatolin A (1)			Obovatolin B (2)		
Position	δ_C_	Type	δ_H_ (*J* in Hz)	δ_C_	Type	δ_H_ (*J* in Hz)
2	82.1	CH	4.80 d (6.3)	82.1	CH	4.79 d (5.6)
3	68.4	CH	4.10 m	68.4	CH	4.07 m
4a	30.3	CH_2_	2.83 dd (16.4, 5.0)	32.8	CH_2_	2.91 dd (16.4, 4.7)
4b			2.66 dd (16.4, 6.8)			2.77 dd (16.4, 6.3)
5	130.2	C		130.3	CH	6.88 d (2.2)
6	115.1	CH	6.34 s	133.1	C	
7	143.0	C		131.1	CH	6.84 d (2.2)
8	136.9	C		127.7	C	
9	144.6	C		150.6	C	
10	116.8	C		121.5	C	
1′	131.3	C		131.6	C	
2′	111.2	CH	6.46 d (1.9)	110.7	CH	6.36 d (2.2)
3′	145.6	C		145.3	C	
4′	138.0	C		137.6	C	
5′	148.1	C		147.9	C	
6′	110.4	CH	6.71 d (1.9)	110.0	CH	6.61 d (2.2)
1″	37.4	CH_2_	3.19 d (6.2)	40.49	CH_2_	3.33 overlap
2″	137.8	CH	5.84 m	139.3	CH	5.96 overlap
3″	116.0	CH_2_	4.92 m	115.8	CH_2_	5.05 m
1‴	134.9	C		132.1	C	
2‴	130.5	CH	7.06 d (8.6)	132.5	CH	6.70 d (2.0)
3‴	117.7	CH	6.78 d (8.6)	127.0	C	
4‴	158.1	C		153.7	C	
5‴	117.7	CH	6.78 d (8.6)	116.8	CH	6.72 d (8.2)
6‴	130.5	CH	7.06 d (8.6)	129.5	CH	6.91 dd (8.2, 2.0)
7‴	40.41	CH_2_	3.32 overlap	40.4	CH_2_	3.20 d (6.8)
8‴	139.1	CH	5.94 overlap	139.6	CH	5.89 m
9‴	115.8	CH_2_	4.98 m	115.4	CH_2_	4.98 m
1″″	135.5	C		135.3	C	
2″″	130.6	CH	7.08 d (8.6)	130.6	CH	7.04 d (8.5)
3″″	118.4	CH	6.86 d (8.6)	118.3	CH	6.76 d (8.5)
4″″	157.5	C		157.4	C	
5″″	118.4	CH	6.86 d (8.6)	118.3	CH	6.76 d (8.5)
6″″	130.6	CH	7.08 d (8.6)	130.6	CH	7.04 d (8.5)
7″″	40.39	CH_2_	3.32 overlap	40.46	CH_2_	3.33 overlap
8″″	139.2	CH	5.93 overlap	139.1	CH	5.95 overlap
9″″	115.7	CH_2_	5.03 m	115.7	CH_2_	5.04 m

## Data Availability

Publicly available datasets were analyzed in this study. This data can be found here: [https://gnps.ucsd.edu/ProteoSAFe/status.jsp?task=3a3d98903cbc4cc5bbb214fa0c7d1ce4], [https://proteomics2.ucsd.edu/ProteoSAFe/status.jsp?task=680053703c2b49f7ab438882c6badab1], [http://string-db.org/], and [http://biosig.unimelb.edu.au/pkcsm/prediction_single/adme_1615762956.82] (accessed on 19 March 2021).

## Supplementary Materials

The following are available online at https://www.mdpi.com/2218-273X/11/3/463/s1, Detailed in silico annotations, IR, NMR, HRMS, UV, and ECD spectra of **1** and **2**, as well as preliminary results of fractions for PCSK9 inhibitory activity and the qPCR results for some selected genes treated by **1**.

## Abbreviations

LC-MS/MS	Liquid chromatography tandem mass spectrometry
MN	Molecular networking
LDL-C	Low-density lipoprotein cholesterol
PCSK9	Proprotein convertase subtilisin/kexin type 9
LDLR	Low-density lipoprotein receptor
GNPS	Global Natural Products Social Molecular Networking
NAP	Network Annotation Propagation
HRESIMS	High resolution-electrospray ionization-mass spectroscopy
SEM	Standard error of the mean
STRING	Search Tool for the Retrieval of Interacting Genes
ADMET	Absorption, Distribution, Metabolism, Excretion and Toxicity
MFs	Molecular families
VDss	Steady state Volume of Distribution
CYP	Cytochrome P 450 enzyme
