# Supplementary material for: Dilignans with a Chromanol Motif Discovered by Molecular Networking from the Stem Barks of Magnolia obovata and Their Proprotein Convertase Subtilisin/Kexin Type 9 Expression Inhibitory Activity
Source: Biomolecules. 2021 Mar 19;11(3):463. doi: 10.3390/biom11030463 (PMC8003705; doi:10.3390/biom11030463)
Supplement: Supplementary file 1 [file biomolecules-11-00463-s001.pdf]

Supplementary Material

Dilignans with a chromanol motif discovered by molecular networking from the stem barks of *Magnolia obovata* and their proprotein convertase subtilisin/kexin type 9 expression inhibitory activity

Jongmin Ahn[a], Hee-Sung Chae[a], Pisey Pel[a], Young-Mi Kim[a], Young Hee Choi[b], Jinwoong Kim[a], Young-Won Chin*,[a]

[a]College of Pharmacy and Research Institute of Pharmaceutical Sciences, Seoul National University, Seoul 08826, Republic of Korea

[b]College of Pharmacy and Integrated Research Institute for Drug Development, Dongguk University-Seoul, Gyeonggi-do 10326, Republic of Korea

*Corresponding author: Phone: +82-2-880-7859; E-mail: ywchin@snu.ac.kr (Y. -W. Chin)

Table of contents

Figure S 1. Effect of fractions from *M. obovata* extract on PCSK9 in the HepG2 human hepatocellular liver carcinoma cell line ....S**Error! Bookmark not defined.**

Figure S 2. *In silico* dereplication of *M. obovata* extract using Network Annotation Propagation (NAP). ....S**Error! Bookmark not defined.**

Figure S 3. ^{1}H NMR (600 MHz, in MeOD) spectrum of Obovatolin A (**1**)S**Error! Bookmark not defined.**

Figure S 4. ^{13}C NMR Spectrum of Obovatolin A (**1**) (150 MHz, in CD_3OD).............. S**Error! Bookmark not defined.**

Figure S 5. ^{1}H-^{1}H COSY NMR Spectrum of Obovatolin A (**1**) (600 MHz, in CD_3OD)S**Error! Bookmark not defined.**

Figure S 6. HSQC NMR Spectrum of Obovatolin A (**1**) (600 MHz, in CD_3OD) .... S**Error! Bookmark not defined.**

Figure S 7. HMBC NMR Spectrum of Obovatolin A (**1**) (600 MHz, in CD_3OD) .... S**Error! Bookmark not defined.**

Figure S 8. ROESY NMR Spectrum of Obovatolin A (**1**) (600 MHz, in CD_3OD)....... S**Error! Bookmark not defined.**

Figure S 9. HRESIMS of Obovatolin A (**1**)..........................S**Error! Bookmark not defined.**

Figure S 10. UV and ECD Spectra of Obovatolin A (**1**) ....S**Error! Bookmark not defined.**

Figure S 11. ^{1}H NMR Spectrum of Obovatolin B (**2**) (600 MHz, in CD_3OD)............. S**Error! Bookmark not defined.**

Figure S 12. ^{13}C NMR Spectrum of Obovatolin B (**2**) (150 MHz, in CD_3OD)............ S**Error! Bookmark not defined.**

Figure S 13. ^{1}H-^{1}H COSY NMR Spectrum of Obovatolin B (**2**) (600 MHz, in CD_3OD)

....S**Error! Bookmark not defined.**

Figure S 14. HSQC NMR Spectrum of Obovatolin B (**2**) (600 MHz, in CD_3OD) .... S**Error! Bookmark not defined.**

Figure S 15. HMBC NMR Spectrum of Obovatolin B (**2**) (600 MHz, in CD_3OD) ...... S**Error! Bookmark not defined.**

Figure S 16. ROESY NMR Spectrum of Obovatolin B (**2**) (600 MHz, in CD_3OD)..... S**Error! Bookmark not defined.**

Figure S 17. HRESIMS of Obovatolin B (**2**).........................S**Error! Bookmark not defined.**

Figure S 18. UV and ECD Spectra of Obovatolin B (**2**)........S**Error! Bookmark not defined.**

Table S 1. Effect of compound 1 on lipid metabolism-related genSe in the HepG2 cells. ...S19

Figure S 19. Pharmacokinetics and drug-likeness prediction for 1 by pkCSM.....................S21

Figure S 1. Effect of fractions from *M. obovata* extract on PCSK9 in the HepG2 human hepatocellular liver carcinoma cell line. Expression of PCSK9 mRNA was assayed by qRT-PCR in cells treated with fractions.

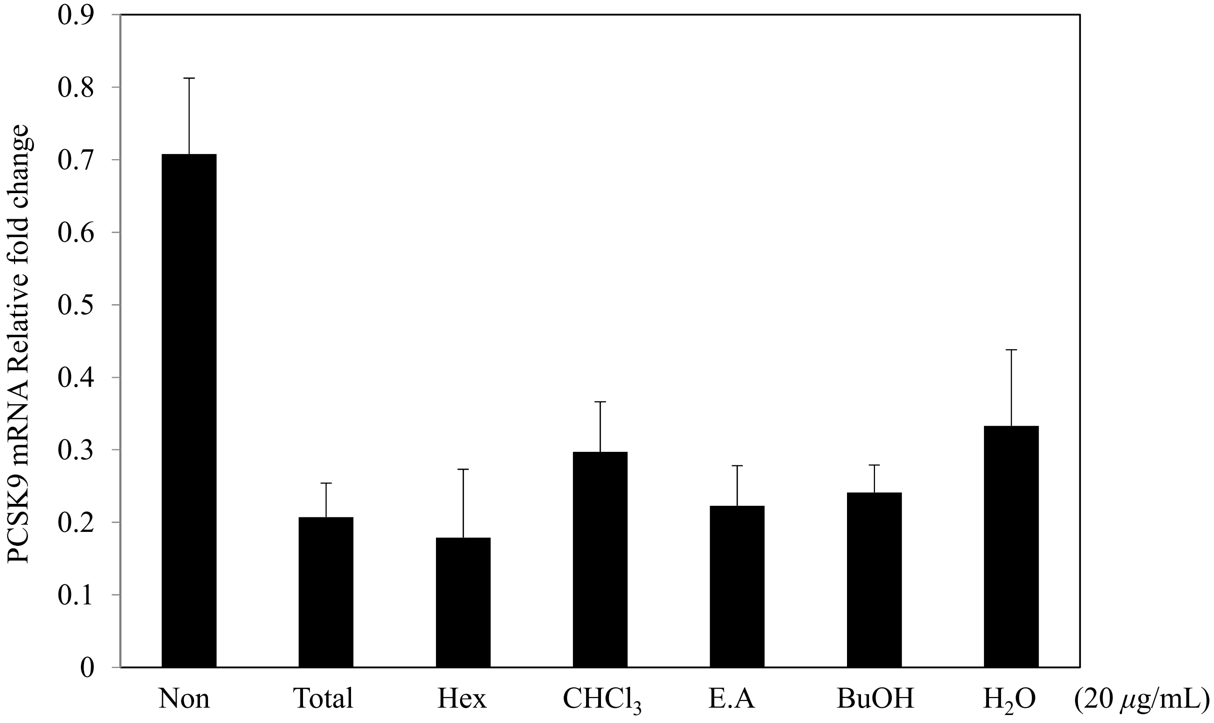

Figure S 2. *In silico* dereplication of *M. obovata* extract using Network Annotation Propagation (NAP). Chemical structures show the most possible candidates predicted by MetFrag; Detailed information on fragmentation pattern analysis can be found at: https://proteomics2.ucsd.edu/ProteoSAFe/status.jsp?task=680053703c2b49f7ab438882c6badab1

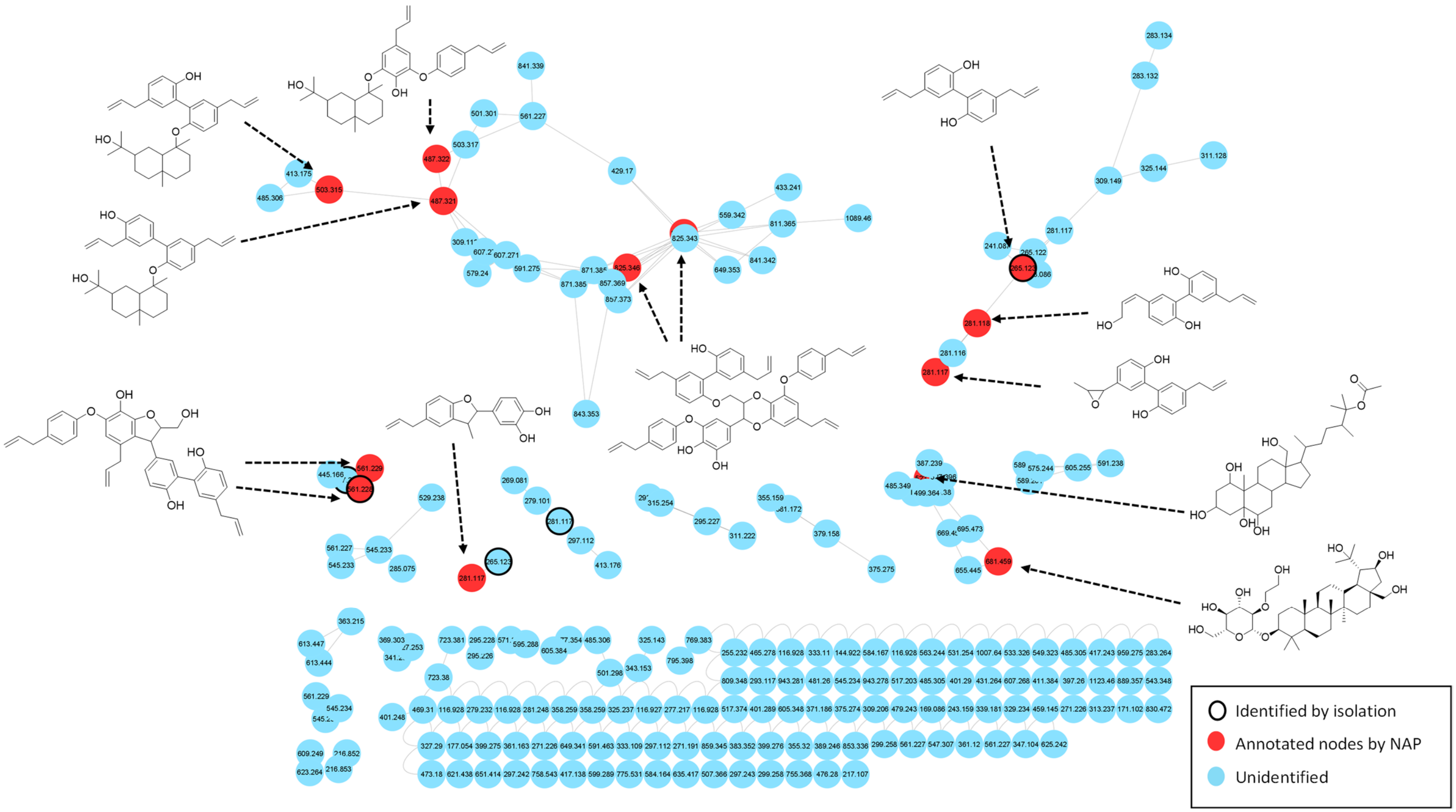

Figure S 3. ^{1}H NMR (600 MHz, in MeOD) spectrum of Obovatolin A (**1**)

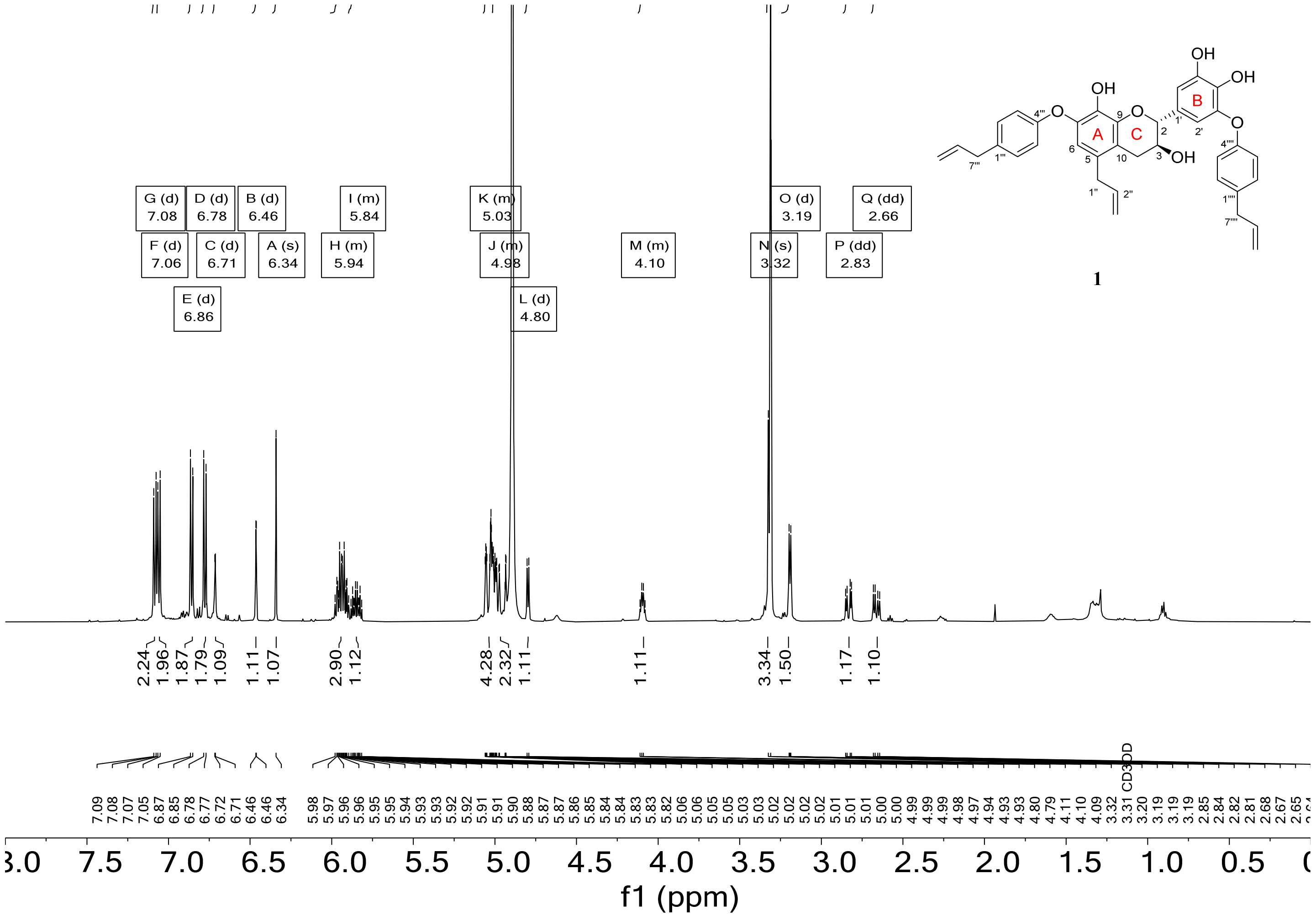

Figure S 4. ^{13}C NMR Spectrum of Obovatolin A (**1**) (150 MHz, in CD_3OD)

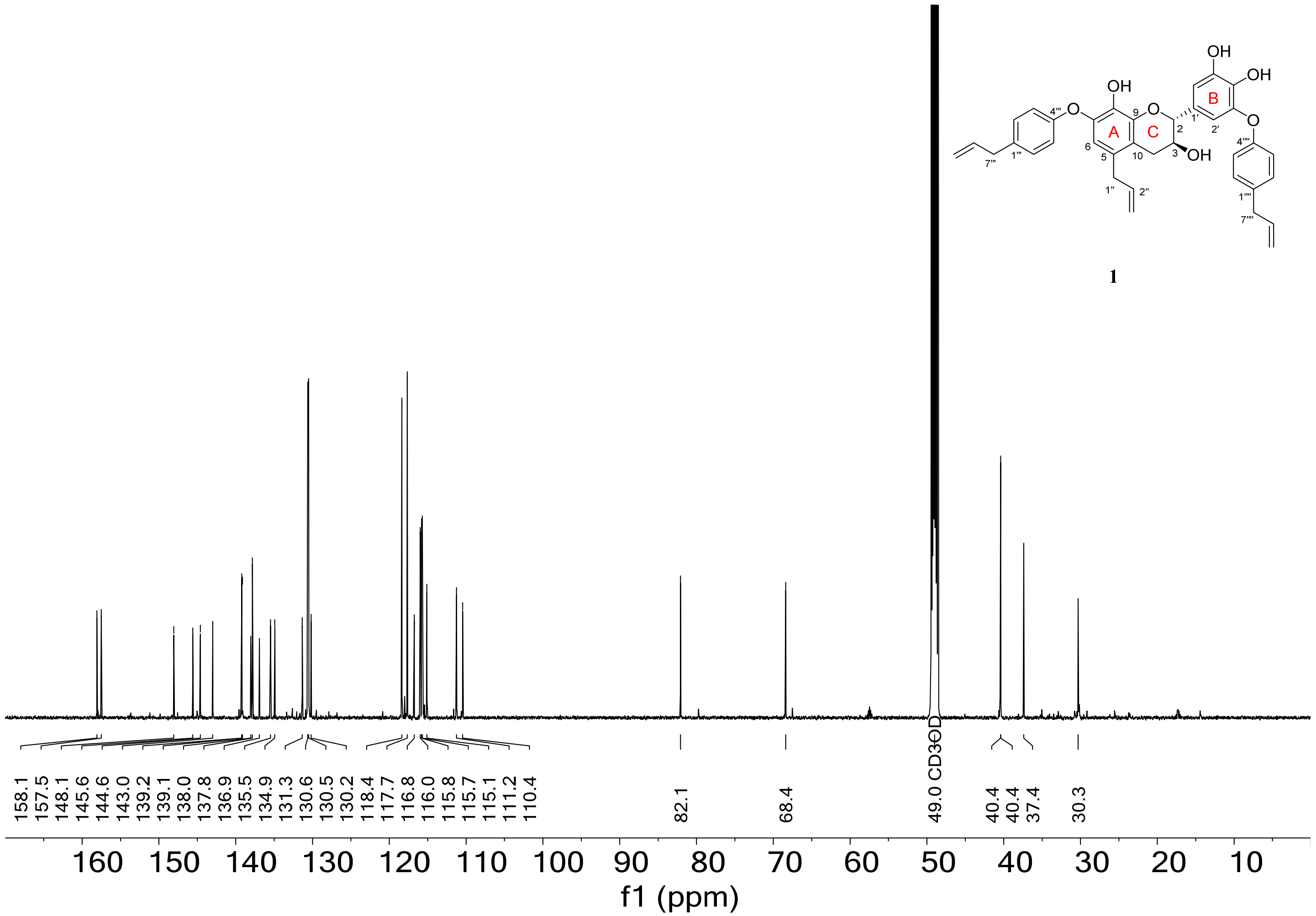

Figure S 5. ^{1}H-^{1}H COSY NMR Spectrum of Obovatolin A (**1**) (600 MHz, in CD_3OD)

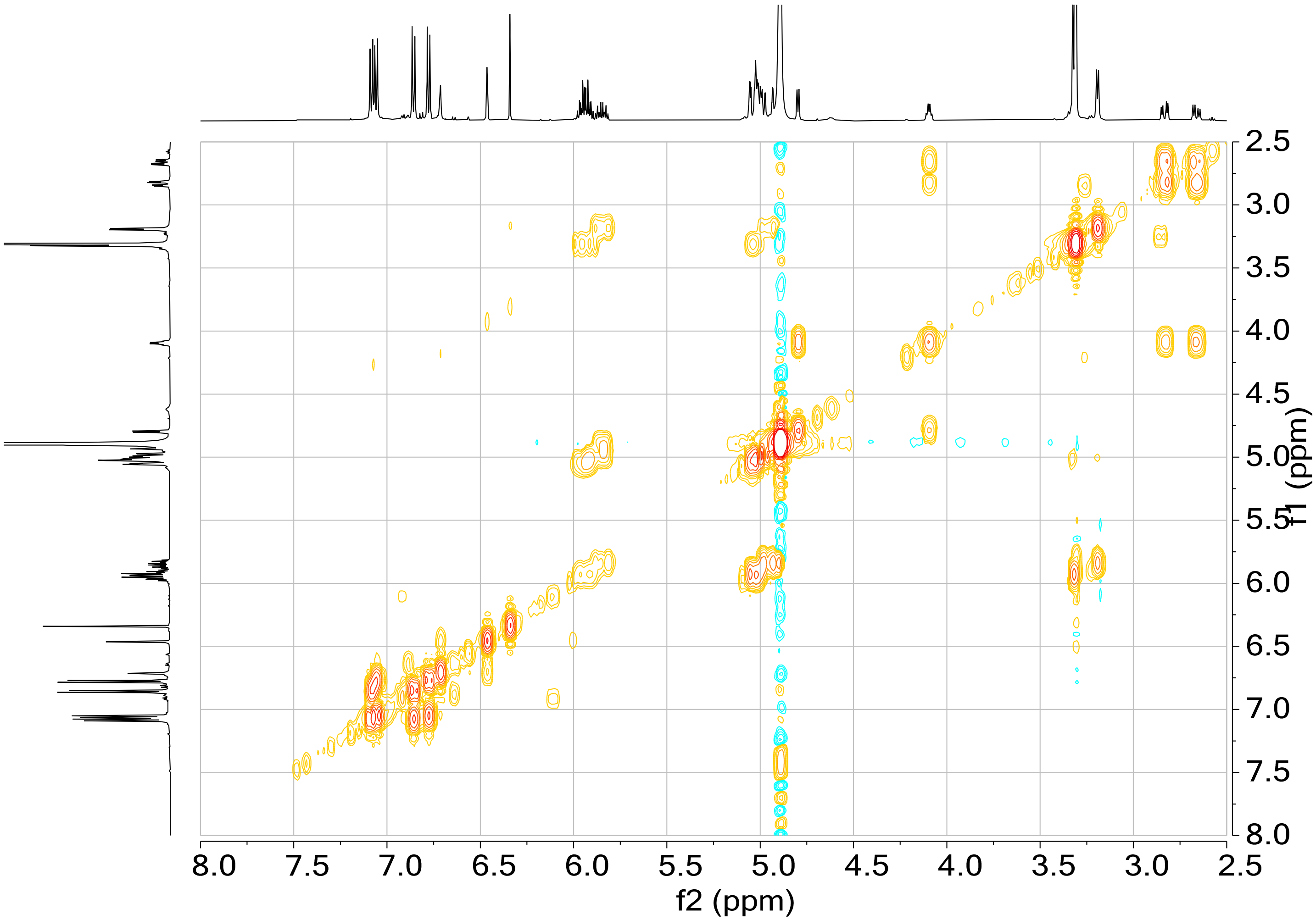

Figure S 6. HSQC NMR Spectrum of Obovatolin A (**1**) (600 MHz, in CD_3OD)

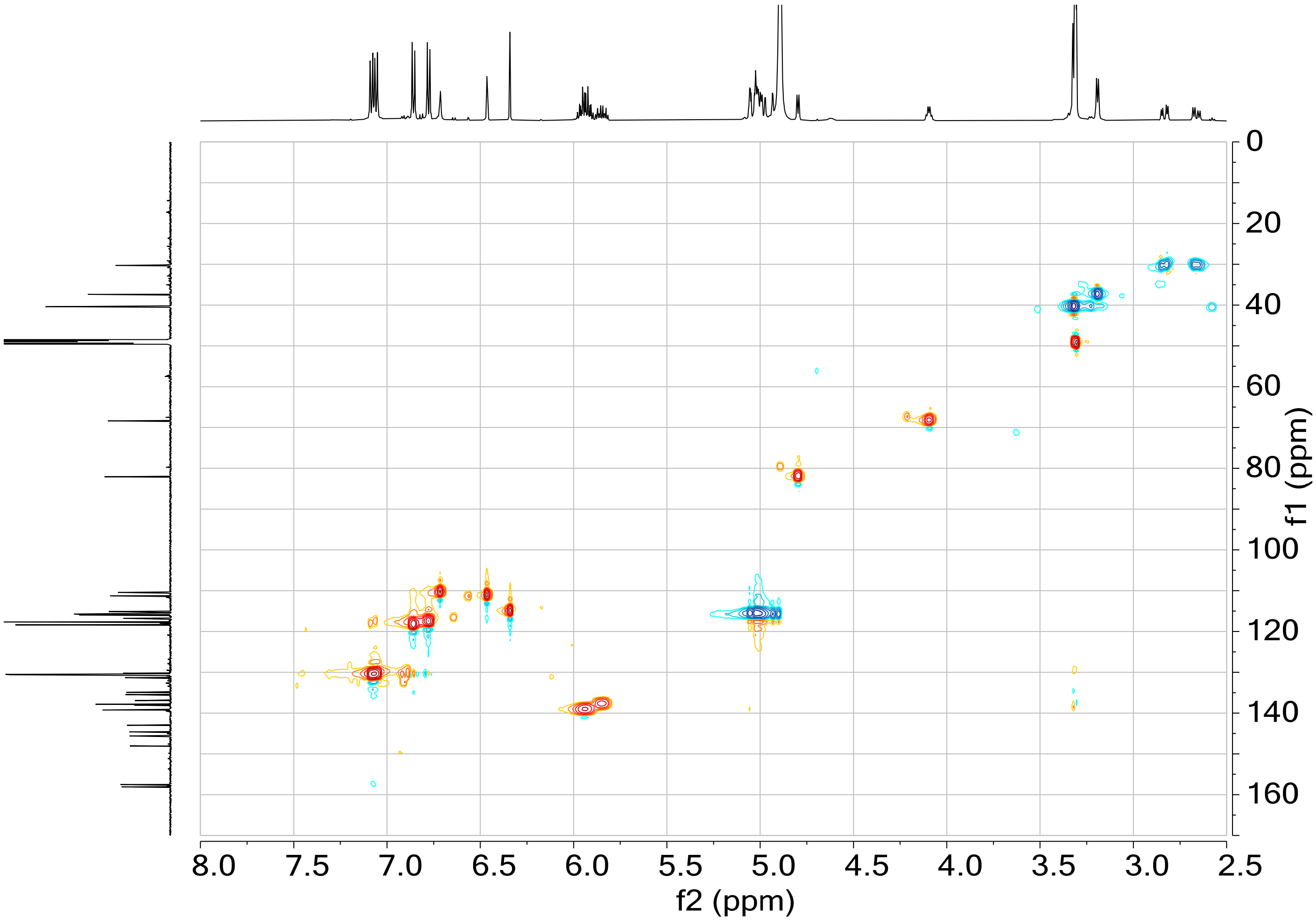

Figure S 7. HMBC NMR Spectrum of Obovatolin A (**1**) (600 MHz, in CD_3OD)

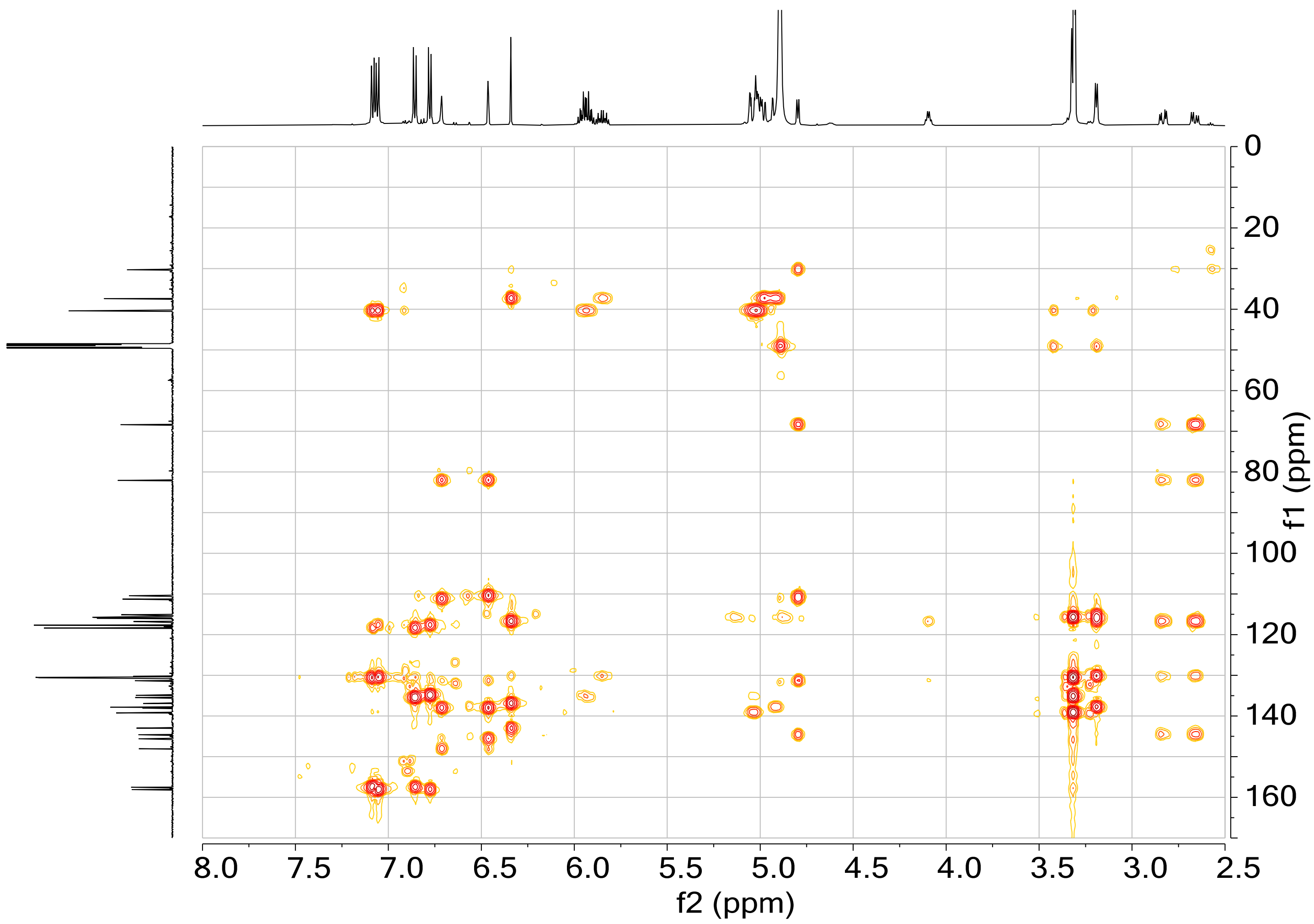

Figure S 8. ROESY NMR Spectrum of Obovatolin A (**1**) (600 MHz, in CD_3OD)

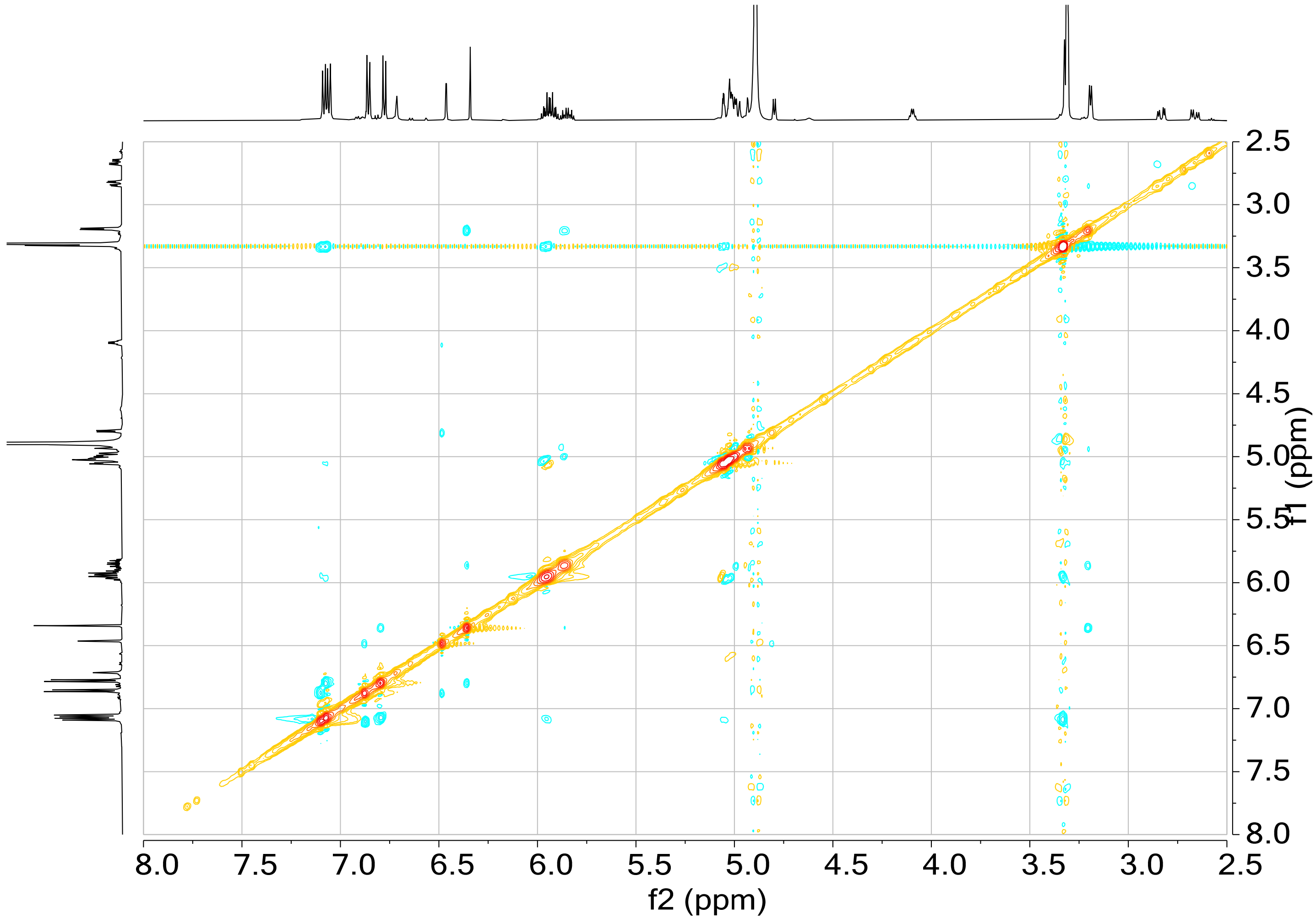

Figure S 9. HRESIMS of Obovatolin A (**1**)

Single Mass Analysis
Tolerance = 5.0 mDa / DBE: min = -1.5, max = 50.0
Element prediction: Off
Number of isotope peaks used for i-FIT = 3
Monoisotopic Mass, Even Electron Ions
162 formula(e) evaluated with 2 results within limits (up to 50 best isotopic matches for each mass)
Elements Used:

Mass	Calc. Mass	mDa	PPM	DBE	Formula	i-FIT	i-FIT Norm	Fit Conf %	C	H	O
577.2233	577.2226	0.7	1.2	20.5	C36 H33 O7	100.8	0.005	99.46	36	33	7
	577.2191	4.2	7.3	-1.5	C18 H41 O20	106.0	5.217	0.54	18	41	20

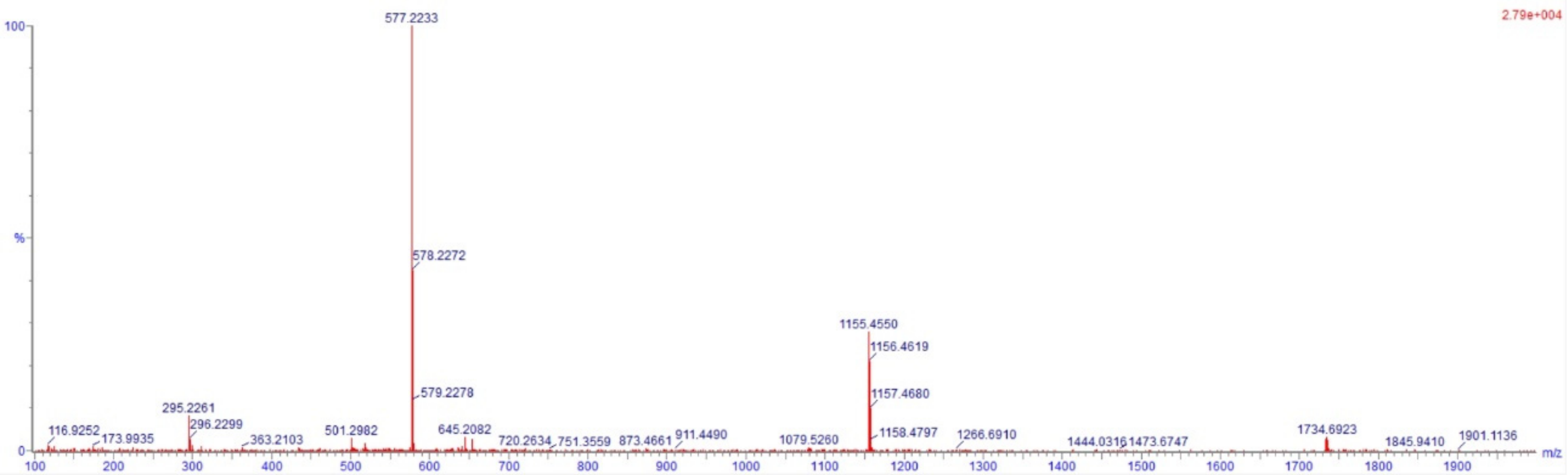

Figure S 10. UV and ECD Spectra of Obovatolin A (**1**)

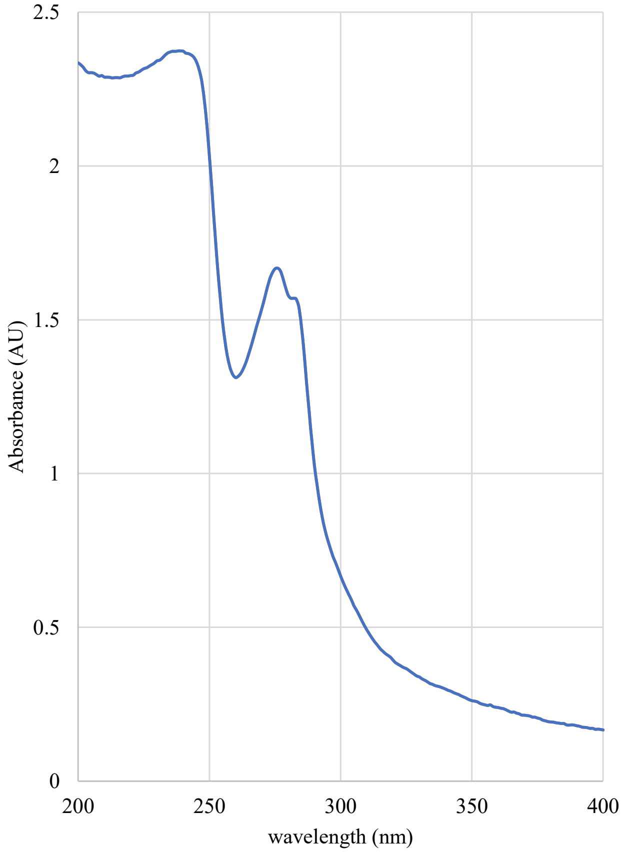

UV Spectrum

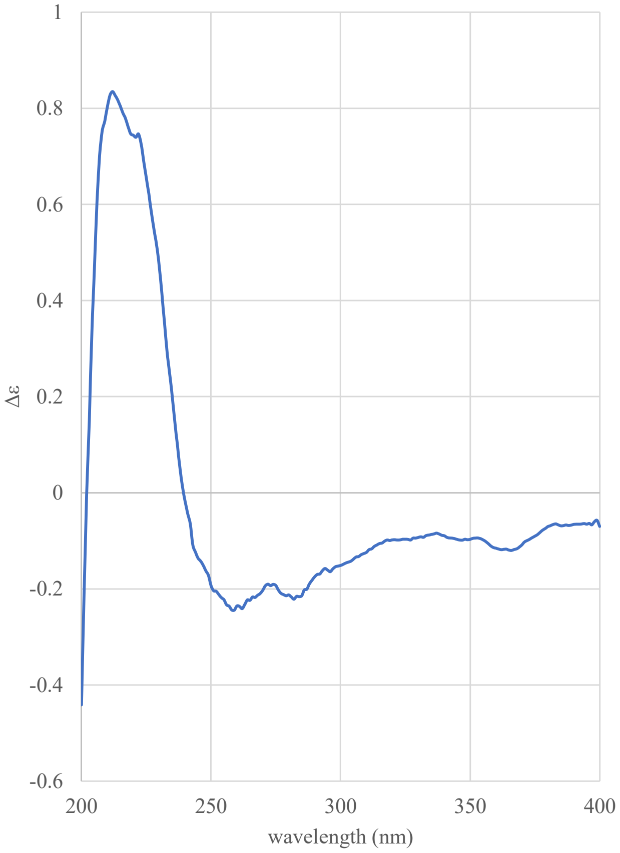

ECD Spectrum

Figure S 11. ^{1}H NMR Spectrum of Obovatolin B (**2**) (600 MHz, in CD_3OD)

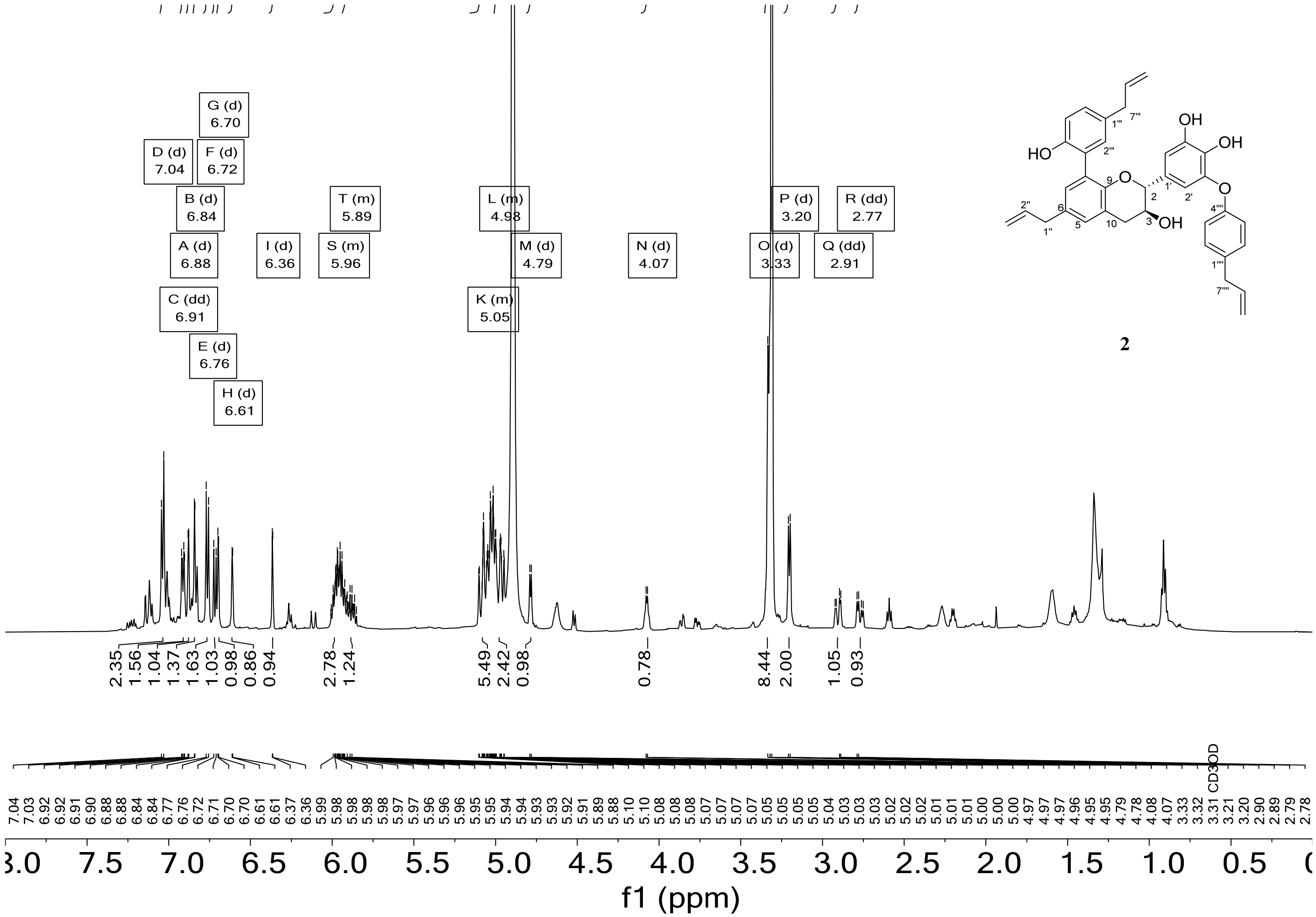

Figure S 12. ^{13}C NMR Spectrum of Obovatolin B (**2**) (150 MHz, in CD_3OD)

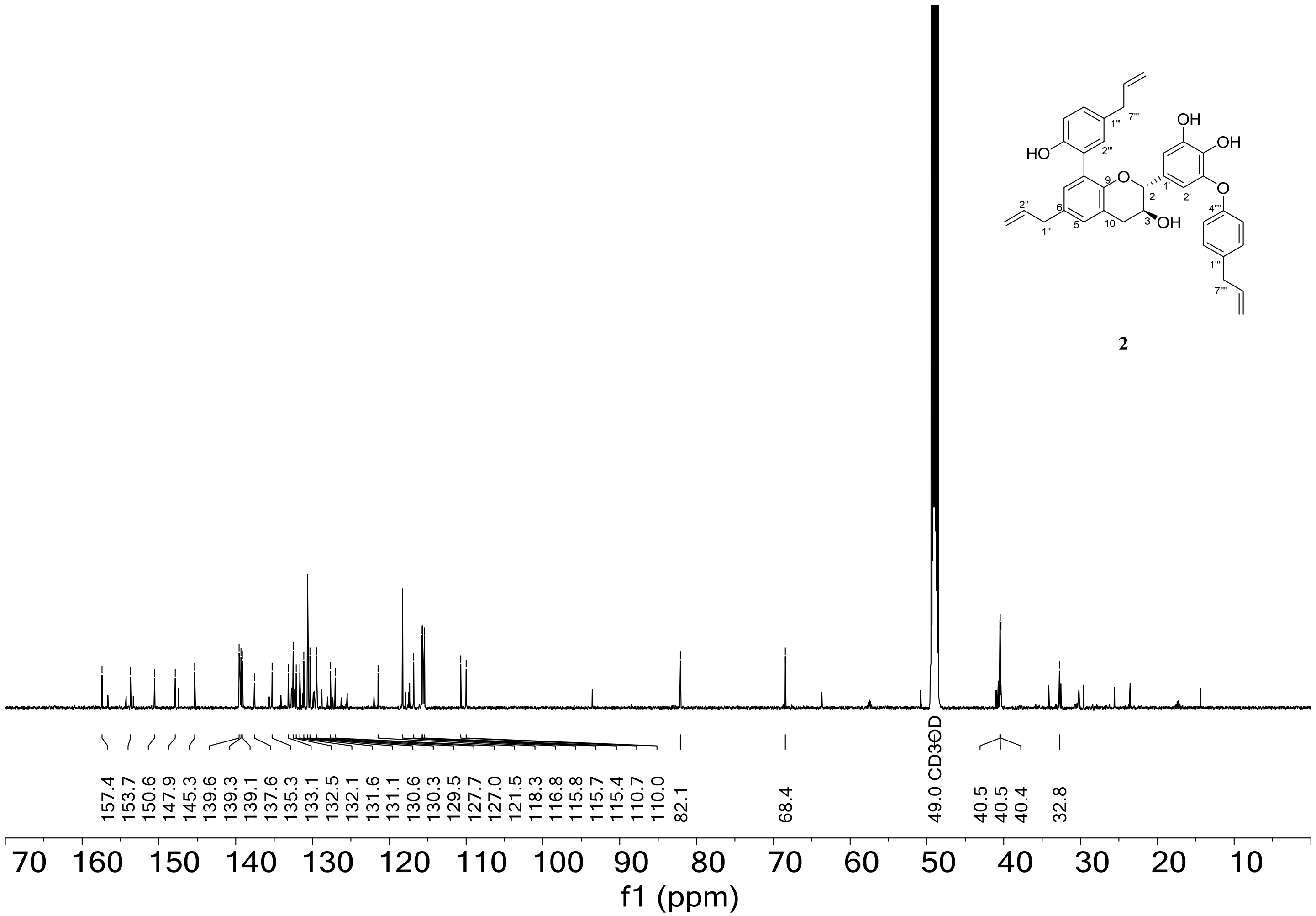

Figure S 13. ^{1}H-^{1}H COSY NMR Spectrum of Obovatolin B (**2**) (600 MHz, in CD_3OD)

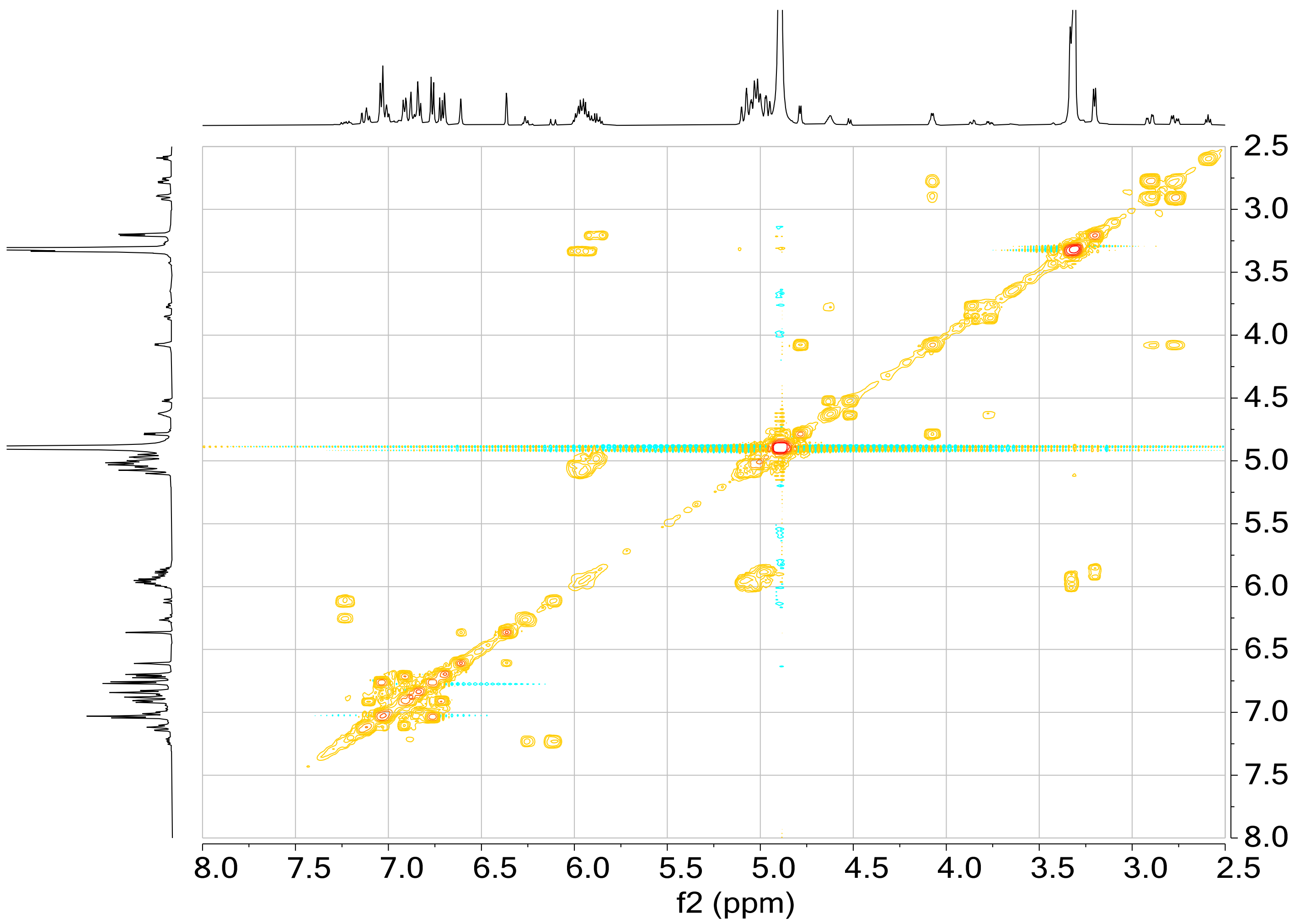

Figure S 14. HSQC NMR Spectrum of Obovatolin B (**2**) (600 MHz, in CD_3OD)

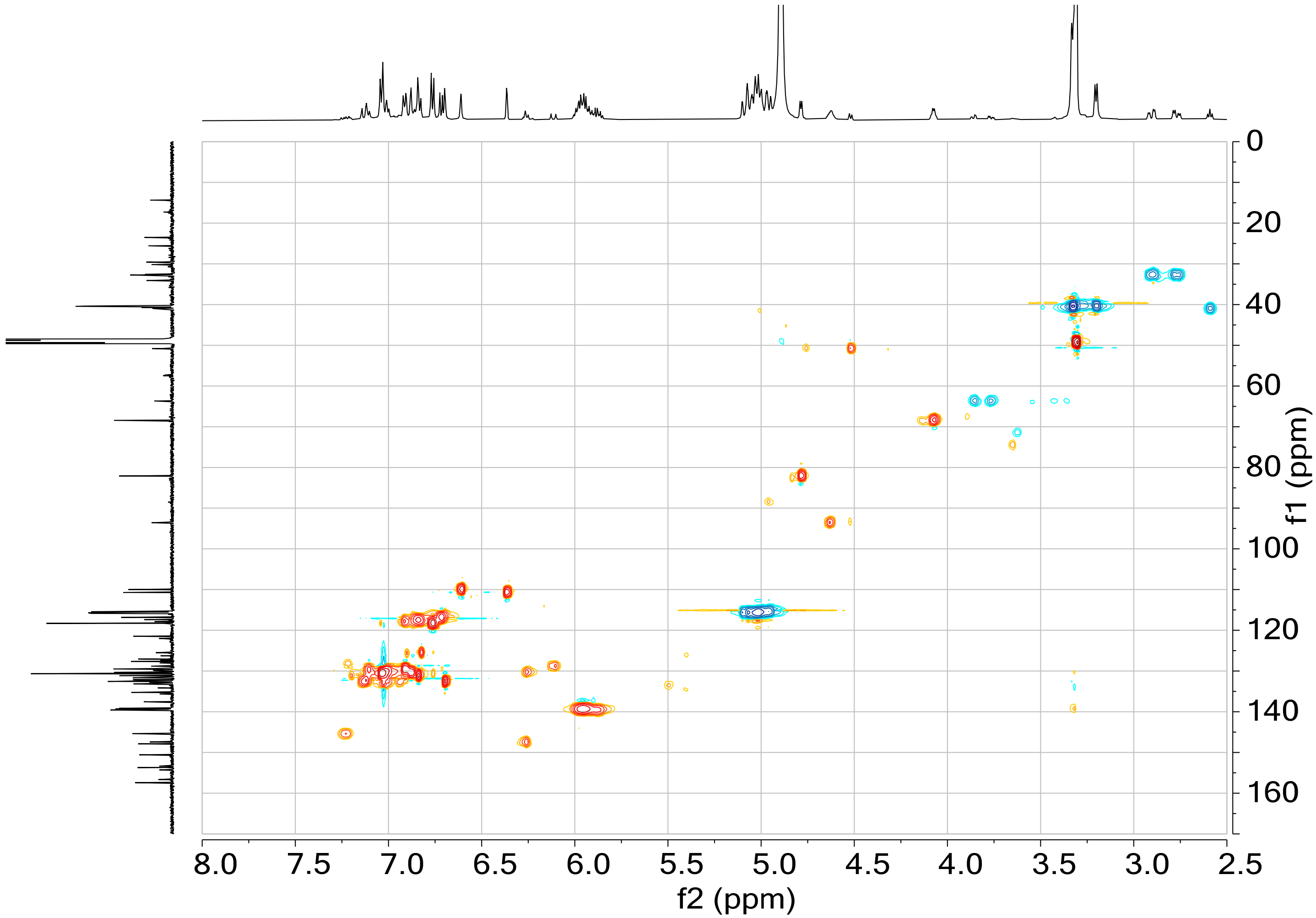

Figure S 15. HMBC NMR Spectrum of Obovatolin B (**2**) (600 MHz, in CD_3OD)

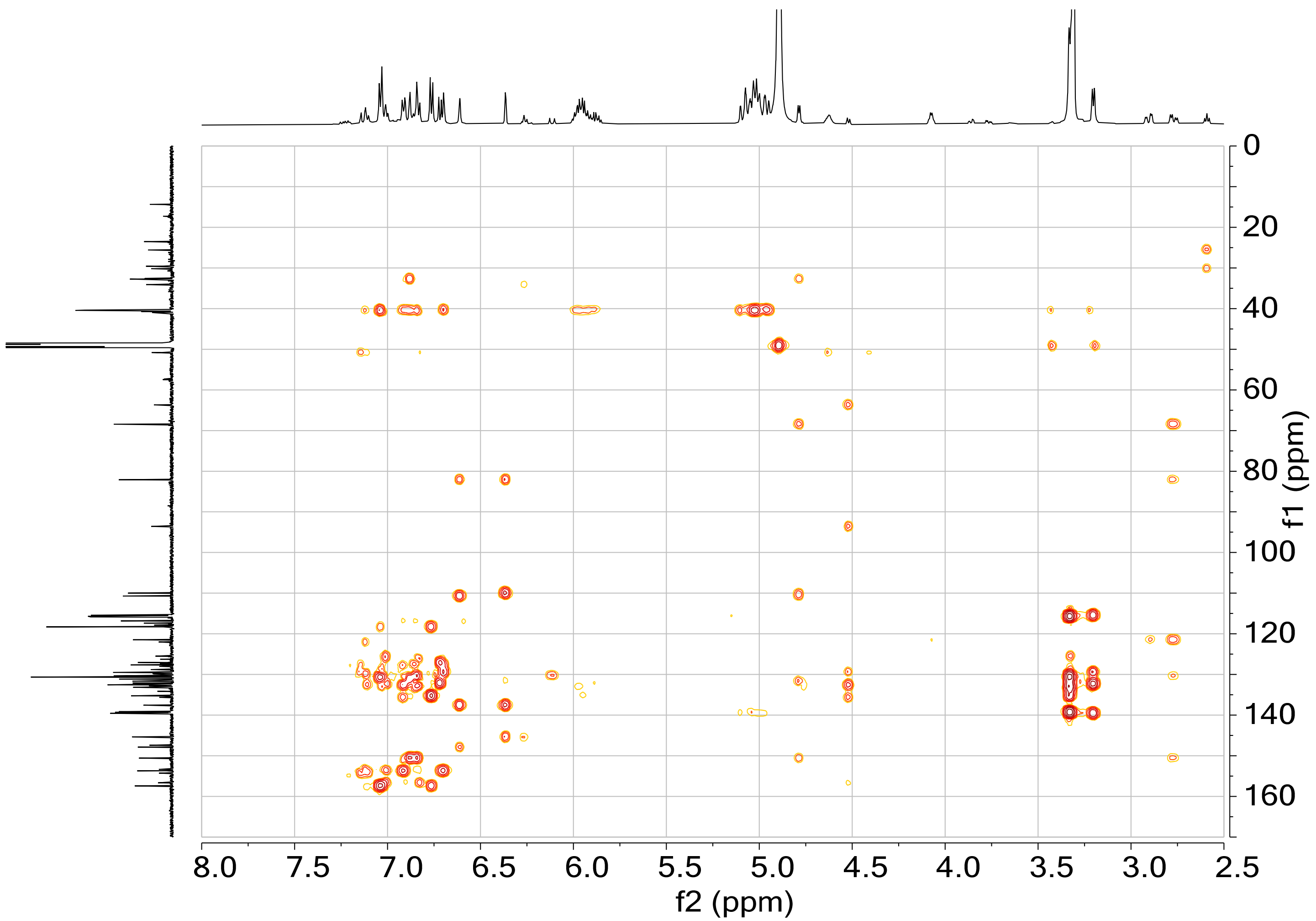

Figure S 16. ROESY NMR Spectrum of Obovatolin B (**2**) (600 MHz, in CD_3OD)

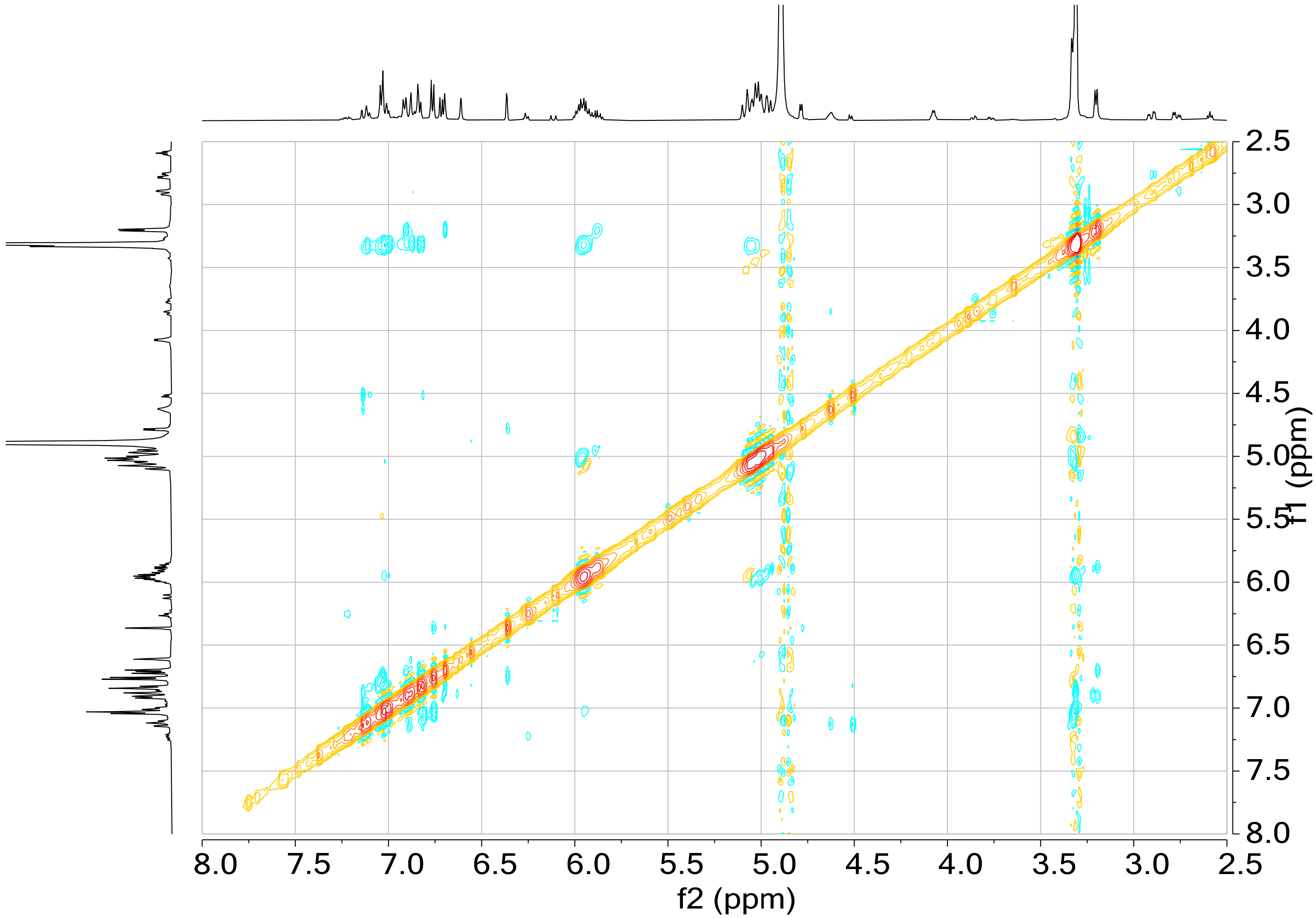

Figure S 17. HRESIMS of Obovatolin B (**2**)

Single Mass Analysis
Tolerance = 5.0 mDa / DBE: min = -1.5, max = 50.0
Element prediction: Off
Number of isotope peaks used for i-FIT = 3
Monoisotopic Mass, Even Electron Ions
154 formula(e) evaluated with 3 results within limits (up to 50 best isotopic matches for each mass)
Elements Used:

Mass	Calc. Mass	mDa	PPM	DBE	Formula	i-FIT	i-FIT Norm	Fit Conf %	C	H	O
561.2257	561.2218	3.9	6.9	29.5	C43 H29 O	144.1	0.372	68.93	43	29	1
	561.2277	-2.0	-3.6	20.5	C36 H33 O6	144.9	1.169	31.07	36	33	6
	561.2242	1.5	2.7	-1.5	C18 H41 O19	153.2	9.456	0.01	18	41	19

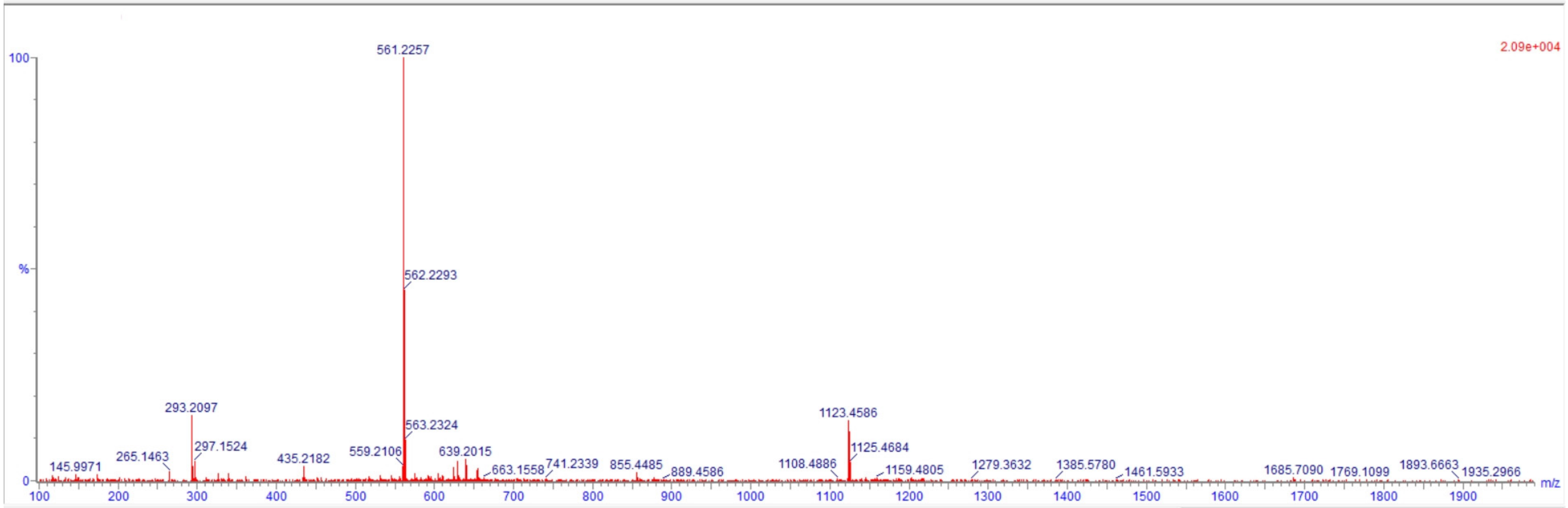

Figure S 18. UV and ECD Spectra of Obovatolin B (**2**)

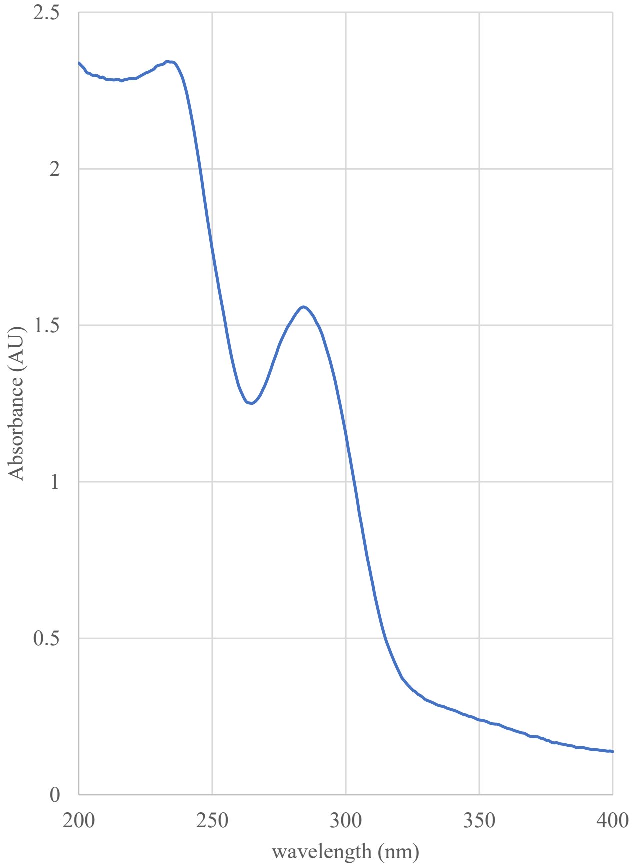

UV Spectrum

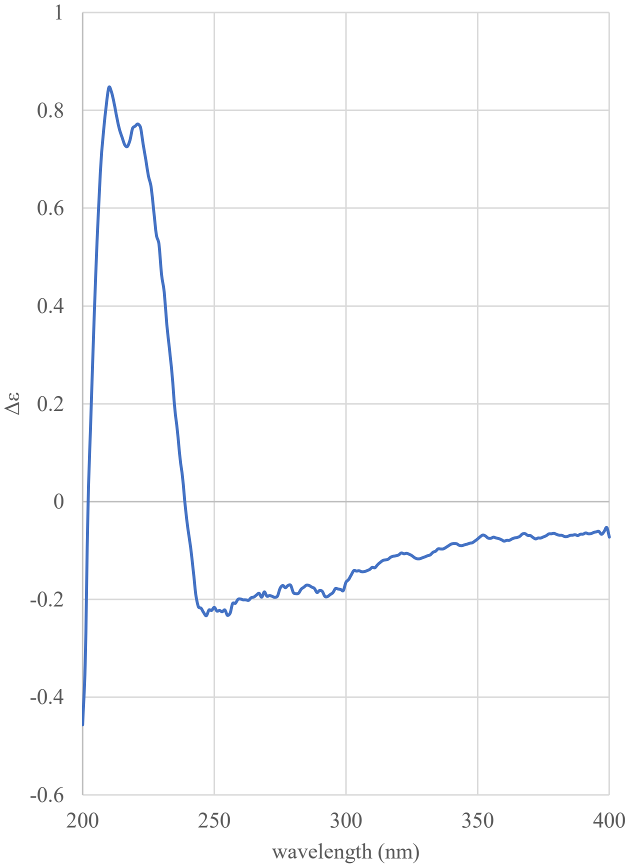

ECD Spectrum

Table S 1. Effect of compound 1 on lipid metabolism-related gene in the HepG2 cells.

Expression of mRNA was assayed by qRT-PCR in cells (significant P value: $p < 0.05$)

Target	Fold Change	P-Value	LEFT PRIMER	RIGHT PRIMER
ABCA1	1.03	0.6113	GTGTTGTCAAGGAGGGGAGA	GCCATCCTAGTGCAAAGAGC
ACSL6	-1.50	0.0038	GAACTACTGGGCCTGCAAAG	TCCGATGTCTCCAGTGTGAA
APOA1	-1.01	0.8458	GAAAGCTGCGGTGCTGAC	TACACAGTGGCCAGGTCCTT
APOA2	1.23	0.1244	GAGAAGGTCAAGAGCCCAGA	TGTGTTCCAAGTTCCACGAA
APOA4	-1.14	0.0211	GGAACAGCTCAGGCAGAAAC	ACCTTGTCCCTCAGGTCCTT
APOB	1.52	0.0222	GCTCCACAGTTTCCAAGAGG	ATTGGTGCCTGTGTTCCATT
APOC2	1.25	0.0035	CCTCCCAGCTCTGTTTCTTG	GGCTAGGCATCTCATCTTGC
APOC3	1.09	0.2196	CCGGGTACTCCTTGTTGTTG	TGTAACCCTGCATGAAGCTG
APOE	1.31	0.0855	CACTGTCTGAGCAGGTGCAG	TCCAGTTCCGATTTGTAGGC
CEBP	1.29	0.0388	GAGGAGGGGAGAATTCTTGG	CATTTCCAAGGCACAAGGTT
CLTC	1.25	0.0202	CAACAATCGCTGGAAACAGA	GCAGGAGTTCTTCAGCCAAT
CPT1A	1.56	0.0365	CAGCAAGTGGAGCTGTTTGA	ACACACCATAGCCGTCATCA
DAB2	-1.25	0.0072	CAGGAGAATGCAGACCATGA	TTGGTAACTGGCAGGGAAAC
DHCR24	1.12	0.2539	ACCTTCCAAAACGACATCCA	GCTCTGCCTCATTTCCTTTG
DHCR7	1.04	0.5414	CTGGACCCTCATCAACCTGT	AGGTACCAGGTTTCGTTCCA
EGFR	-1.08	0.3067	GCCCTGATGGATGAAGAAGA	GGAATTGTTGCTGGTTGCAC
FDPS	-1.16	0.0342	GGAGATGGGGGAGTTCTTTC	GTCCCCAAAGAGGTCAAGGT
FOXA2	-1.32	0.2841	ATGCACTCGGCTTCCAGTAT	GTTGCTCACGGAGGAGTAGC
FZD4	-1.23	0.2999	CCTGGCCAGAGAGTCTGAAC	GTTGTGGTCGTTCTGTGGTG
HIF1A	1.19	0.0396	TGCTGAAGACACAGAAGCAAA	TGGTGACAACTGATCGAAGG
HMGCR	-1.10	0.1487	AAACATTGTCACCGCCATCT	GGGACCACTTGCTTCCATTA
HNF1A	-1.12	0.6450	CGCAGACTATGCTCATCACC	CTGGAGGCCTCAGTGTCTG
KLF6	1.24	0.2014	TCGGGAGAAAAAGGAGGAAT	AAAGTTCCTCGGAGCTGTCA
LDLR	-1.09	0.4142	GACGTGGCGTGAACATCTG	CTGGCAGGCAATGCTTTGG
LDLRAP1	1.51	0.0681	AAGACTGCACCCCCTCCTT	GGGCCTTAGCTGTCTCCTCT
LIPC	1.14	0.0142	CCCACGACCACTACACCATC	ACCCAGGCTGTACCCAATTA
NR1H3	1.33	0.0140	CTGCCCAGCAACAGTGTAAC	GATGGCCAGCTCAGTGAAGT
MyLIP	-1.26	0.0255	GAAACTGCTCATTGGGGTTG	TTCCTTGGTGACCGTCAAAT
NANOG	-1.12	0.6180	TACCTCAGCCTCCAGCAGAT	TTGCTATTCTTCGGCCAGTT
PCSK9	-1.89	0.0321	AGGGGAGGACATCATTGGTG	CAGGTTGGGGGTCAGTACC
PPARa	1.31	0.0305	ATGGCATCCAGAACAAGGAG	GGCGAATATGGCCTCATAAA
PPARr	-1.06	0.4150	GCTGGCCTCCTTGATGAATA	TTGGGCTCCATAAAGTCACC
NR1I2	-1.34	0.1225	GAGACTGGAACCTGGGAGTG	TGGGCTCCAGTAGAAGTTGC

RXRa	1.33	0.1403	ACATGGCTTCCTTCACCAAG	GGTGTAGGTCAGGTCCTTGC
SCAP	-1.59	0.2731	CATGGAGACGTCACGCTGTA	GTAGAGGCAGAGCAGCAGCA
SP4	-1.22	0.2421	AATGGAATGCAGAATGCACA	ACGACTGTGGTGGAATAGCC
SQLE	1.08	0.1433	GGAAAAGCCTGGTCTCCAAT	GAGAACTGGACTCGGGTTAGC
SREBF1	-1.13	0.2303	CCTTGCATTTTCTGACACGCT	TCCCCATCCACGAAGAAACG
SREBF2	-1.16	0.1460	GACGCCAAGATGCACAAGTC	ACCAGACTGCCTAGGTCGAT
STON2	-1.01	0.9493	CCACCAGTCAGAATGGGTCT	GACTGGTCTGGGGAAGATGA
TFRC	1.35	0.0101	ATCCGGTTACTGGGCAATTT	AAGGAAAGGGAAAGCAGCAT
TM7SF2	-1.04	0.6155	GCGAATTCCCAGAAAAACAC	ACCCAGACACCAGCAGTTTC
GAPDH	-	-	ATGGGGAAGGTGAAGGTCG	GGGGTCATTGATGGCAACAATA

Figure S 19. Pharmacokinetics and drug-likeness prediction for **1** by pkCSM.

Molecule Depiction

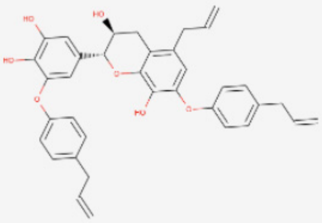

SMILES

Molecule properties:

Descriptor	Value
Molecular Weight	578.661
LogP	7.6106
#Rotatable Bonds	11
#Acceptors	7
#Donors	4
Surface Area	250.425

Property	Model Name	Predicted Value	Unit
Absorption	Water solubility	-3.037	Numeric (log mol/L)
Absorption	Caco2 permeability	0.408	Numeric (log Papp in 10^{-6} cm/s)
Absorption	Intestinal absorption (human)	87.173	Numeric (% Absorbed)
Absorption	Skin Permeability	-2.735	Numeric (log Kp)
Absorption	P-glycoprotein substrate	No	Categorical (Yes/No)
Absorption	P-glycoprotein I inhibitor	Yes	Categorical (Yes/No)
Absorption	P-glycoprotein II inhibitor	Yes	Categorical (Yes/No)
Distribution	VDss (human)	-1.251	Numeric (log L/kg)
Distribution	Fraction unbound (human)	0.391	Numeric (Fu)
Distribution	BBB permeability	-1.219	Numeric (log BB)
Distribution	CNS permeability	-2.7	Numeric (log PS)
Metabolism	CYP2D6 substrate	No	Categorical (Yes/No)
Metabolism	CYP3A4 substrate	Yes	Categorical (Yes/No)
Metabolism	CYP1A2 inhibitior	No	Categorical (Yes/No)
Metabolism	CYP2C19 inhibitior	No	Categorical (Yes/No)
Metabolism	CYP2C9 inhibitior	Yes	Categorical (Yes/No)
Metabolism	CYP2D6 inhibitior	No	Categorical (Yes/No)
Metabolism	CYP3A4 inhibitior	Yes	Categorical (Yes/No)
Excretion	Total Clearance	0.44	Numeric (log ml/min/kg)
Excretion	Renal OCT2 substrate	No	Categorical (Yes/No)
Toxicity	AMES toxicity	No	Categorical (Yes/No)
Toxicity	Max. tolerated dose (human)	0.221	Numeric (log mg/kg/day)
Toxicity	hERG I inhibitor	No	Categorical (Yes/No)
Toxicity	hERG II inhibitor	Yes	Categorical (Yes/No)
Toxicity	Oral Rat Acute Toxicity (LD50)	2.523	Numeric (mol/kg)
Toxicity	Oral Rat Chronic Toxicity (LOAEL)	2.429	Numeric (log mg/kg_bw/day)
Toxicity	Hepatotoxicity	No	Categorical (Yes/No)
Toxicity	Skin Sensitisation	No	Categorical (Yes/No)
Toxicity	*T.Pyriformis* toxicity	0.285	Numeric (log ug/L)
Toxicity	Minnow toxicity	-2.072	Numeric (log mM)
